# Metabolic traits of sediment bacteria in karst caves in the light of environmental changes

**DOI:** 10.3389/fmicb.2025.1724116

**Published:** 2025-12-12

**Authors:** Janez Mulec, Lejla Pašić, Andreea Oarga-Mulec

**Affiliations:** 1 Karst Research Institute, Research Centre of the Slovenian Academy of Sciences and Arts, Postojna, Slovenia; 2 UNESCO Chair on Karst Education, University of Nova Gorica, Vipava, Slovenia; 3 Postgraduate School, Research Centre of the Slovenian Academy of Sciences and Arts, Ljubljana, Slovenia; 4 Sarajevo Medical School, University Sarajevo School of Science and Technology, Sarajevo, Bosnia and Herzegovina; 5 Materials Research Laboratory, University of Nova Gorica, Nova Gorica, Slovenia

**Keywords:** 16S rRNA gene amplicon sequencing, cave sediment microbiome, community-level physiological profiling, diversity, ecosystem resilience

## Abstract

**Introduction:**

Karst subterranean systems are vulnerable ecosystems that have not yet been studied adequately at the microbial functional level. Cave sediments deposited over different time periods host diverse microbial communities that play a critical role in nutrient cycling and pollutant degradation.

**Methods:**

In this study, we investigated microbial diversity and metabolic capacity in recently deposited alluvial sediments and an ancient palaeo-river deposit in a karst cave system. Using 16S rRNA gene amplicon metagenomic analysis, community-level physiological profiling (CLPP), and chemical characteristics of the environment, the influence of key environmental factors on microbial community composition and substrate degradation, concentrating particularly upon sediment age, oxygen availability, and temperature, was assessed.

**Results:**

The results showed different microbiome compositions and metabolic characteristics between sites. The old alluvial sediment exhibited low taxonomic and functional diversity, accompanied by elevated heavy-metal concentrations, suggesting that sediment age might act as a geochemical filter, limiting microbial function. In contrast, a periodically flooded site showed high metabolic versatility and taxonomic diversity, emphasizing the ecological role of hydrological pulses in maintaining functional microbial diversity. CLPP metrics linked community structure to functional potential, revealing adaptive traits in key taxa such as *Polaromonas*, *Methylibium,* and *Beggiatoa*.

**Discussion:**

These results demonstrated the value of integrating functional and taxonomic approaches in subsurface environments and provide insights into microbial resilience, biogeochemical processes, and the potential for applied environmental use.

## 1 Introduction

Subterranean karst systems play a crucial role in the global water cycle, currently contributing around 25% of the world’s drinking water (Ford and Williams, 2007). Caves intercept the water cycle, with dripping water and underground rivers transporting and depositing both organic and inorganic materials that accumulate and act as long-term integrators of hydrological, chemical, and biological processes (Addesso et al., 2023; Mulec et al., 2019). These fragile environments are particularly sensitive to global climate changes, including alterations in precipitation and temperature regimes (Mammola et al., 2019; Medina et al., 2023), as well as increased pollution and land-use pressures (Mahler et al., 2021; Zhang et al., 2023). They are increasingly exposed to hydroclimatic extremes, including floods, droughts, and rising cave temperatures (Howarth et al., 2025), which all impact subsurface microbial activity and geochemical cycling directly.

Sediments accumulating in the subsurface support dynamic microbial habitats shaped by hydrological and geochemical variability (Thullner and Regnier, 2019), yet their ecological functioning remains underinvestigated in comparison to that in surface environments. Subsurface is usually not quantifiable and is generally defined as the zone below the Earth’s surface, encompassing layers of soil, sediment, and rock beneath the ground. Sediments are valuable markers for assessing sedimentation periods and reconstructing past geological, climatic, and biological events (Evans et al., 2025; Hunt et al., 2015; Kurečić et al., 2021; White, 2007; Zupan Hajna et al., 2008a). Cave sediments not only provide records of environmental influences, but also of microbial adaptations in the face of changing conditions. Microbial communities respond rapidly to variations in nutrient availability, oxygen, and temperature, offering important insights into the resilience of subsurface ecosystems. These communities include diverse taxa (Martin-Pozas et al., 2023). Some of them produce metabolites of biotechnological interest (Bontemps et al., 2024; Chelius et al., 2009; Wiseschart et al., 2019), particularly those capable of pollutant degradation and other bioactive compounds (Meka et al., 2024) and can represent a source of antibiotic resistance genes (ARGs) (Gemeda et al., 2025; Skok et al., 2020). From a metabolic perspective, cave sediments commonly support microbial chemoheterotrophy (Ma et al., 2021) and also lithoautotrophy (Turrini et al., 2024). Diverse microbes in cave environments are active in biogeochemical cycling of nitrogen (ammonia oxidation, nitrogen fixation, denitrification), sulfur (sulfur oxidation, sulfate reduction), iron (iron oxidation, iron reduction), and manganese (oxidation). Methanotroph bacteria and methylotrophs are widespread in cave sediments (Zhu et al., 2022). Biogenic methanogenesis in cave ecosystems is also observed (Schirmack et al., 2014), in different caves, linked potentially to the ubiquitous presence of archaea *Methanosarcina* (Allenby et al., 2022).

Cave sediments are not just passive deposits, but dynamic microbial habitats that influence biogeochemical processes through nitrogen, sulfur, and carbon cycling, and represent also a considerable microbial diversity (Aronson et al., 2023; Kumaresan et al., 2018). A stronger focus is needed on quantifying how microbial communities mediate and respond to elemental cycling, because these feedbacks remain insufficiently characterized in subterranean sediments (Guan et al., 2023). Whereas microbial responses to environmental change have been investigated widely in surface ecosystems, subterranean sediments remain less-well studied, particularly with regard to their functional roles. Most studies rely primarily upon genomic or cultivation-based approaches, whereas techniques such as community-level physiological profiling (CLPP) remain underused. Understanding microbial metabolic versatility and adaptation strategies in these sediments is therefore essential for helping to predict subsurface ecosystem responses within future climate scenarios.

In this study, we examined microbial diversity and metabolic response of microorganisms in recently deposited alluvial cave sediments and in a sediment deposited by a palaeo-river. The experiments were designed to allow observation of the impact of temperature and the presence/absence of oxygen on microbiota, and to support identification of patterns in their metabolic responses. The combination of CLPP with taxonomic profiling presented advances in understanding microbial metabolic flexibility in karst sediments under varying environmental conditions, revealing functional processes that might remain hidden during purely taxonomic studies. This functional profiling is essential to the prediction of how microbial communities mediate and adapt to short-term environmental stressors, especially in habitats where traditional monitoring approaches are limited.

## 2 Materials and methods

### 2.1 Site description

**Figure 1:**
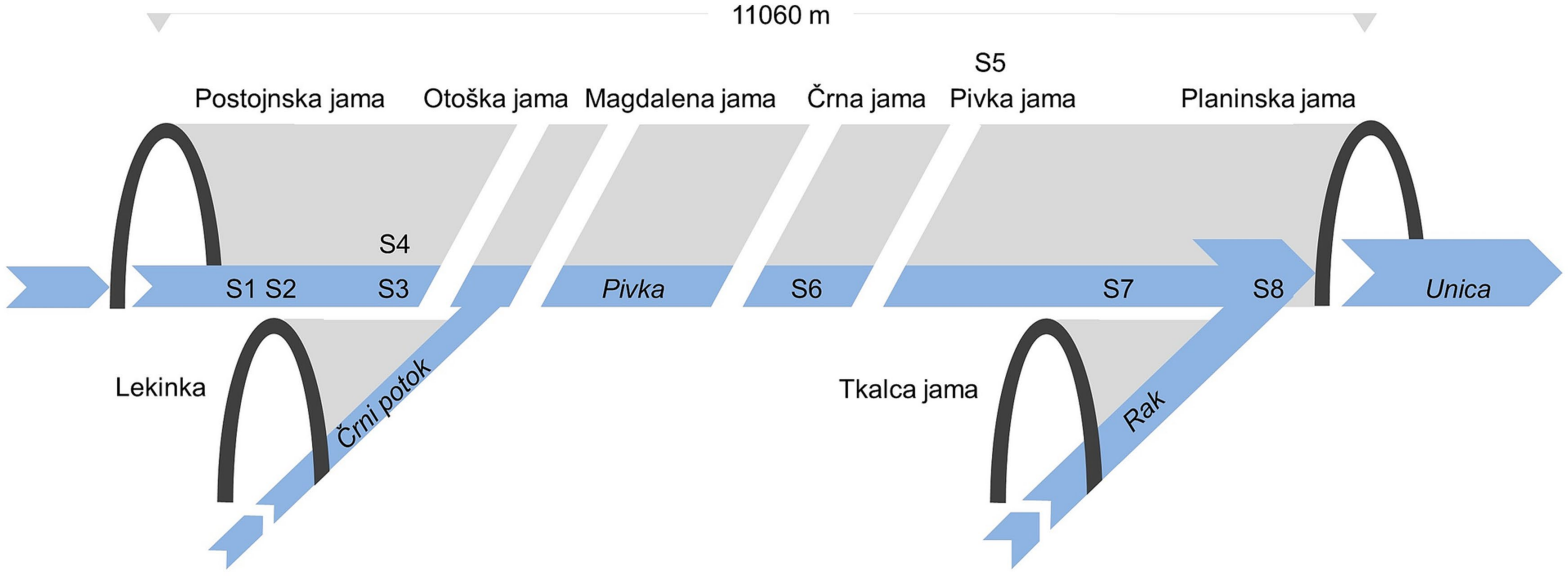
Schematic representation of sampling site relationships in the Postojna–Planina Cave System: S1–S4 in Postojnska jama, S5 karst soil above Pivka jama, S6 in Črna jama, S7 and S8 in Planinska jama. Distance of sampling sites from the ponor at Postojnska jama to: S1/S2–75 m, S3/S4–1,000 m, S6–3,000 m, S7–10,000 m, S8–11,000 m.

The Postojna–Planina Cave System (PPCS)—“Postojnsko–planinski jamski sistem” in Slovene—is a complex underground karst system of several interconnecting dry and submerged passages at two main levels, exceeding 30 km in total length. The PPCS is formed predominantly in a thick sequence of Late Cenomanian and Turonian to Senonian (Cretaceous) limestones (Zupančič et al., 2011; Šebela, 2012). At the upstream limit of the PPCS, the Pivka River sinks into the cave Postojnska jama (Postojna Cave) at 512 m a.s.l. (45.781840° N 14.203966° E). Along its underground course, the river passes through parts of several other caves that also provide access to the system (i.e., Otoška jama, Magdalena jama, Črna jama, Pivka jama). While still underground, the Pivka converges with the Rak River in Planinska jama (Planina Cave). Downstream of the confluence, the river, now named the Unica, flows out from Planinska jama (455 m a.s.l., 45.8157543° N 14.2453978° E). One other minor allogenic recharge for the PPCS is the Črni potok stream, which sinks in the Lekinka cave (514 m a.s.l., 45.788584° N 14.193232° E), and subsequently joins the underground Pivka River (Figure 1; Supplementary Figure S1). The water quality of the Pivka River is impacted by the effects of agriculture and local industry, and it also collects treated water from the municipal wastewater treatment plant serving the Postojna town area (21,000 population equivalent).

Passages and channels within the PPCS contain various alluvial sediment deposits characteristic of the internal cave facies, such as silts, sands, and gravels, covered by, and/or intercalated with layers of speleothem (Zupan Hajna et al., 2008b). Inorganic material, represented mostly by minerals from the eroded flysch bedrock, is co-deposited with particulate and dissolved organic matter (plant and animal debris). Sediments in the riverbed are subjected to deposition–erosion episodes, particularly during extreme hydrological events, and are discontinuously deposited, eroded, and redeposited between the ponor and the spring.

### 2.2 Sediment sampling

Seven sediment samples (600 g per site, depth up to 2 cm) were collected in three caves (Postojnska jama, Črna jama, and Planinska jama) along the underground course of the Pivka River. In addition, a sample of karst soil was collected from the land surface above Pivka jama (Figure 1).

Four samples were collected in Postojnska jama. In the bed of the Pivka River, two samples were taken from a 1.5-m-high sediment pile deposited 75 m from the ponor: S1 at a height of 1 m, and S2 at the foot of the pile. Sample S3 was collected 1,000 m inside the cave, from an occasionally flooded side branch of the Pivka River. Sample S4 was collected in the same chamber (Spodnji Tartar) as S3, from a sediment pile at a higher elevation. This sample originated from an old and partly eroded, 13-m-high pile deposited by the palaeo-river, and is not influenced by the present-day Pivka River. Palaeomagnetic dating of this portion of sediment indicated sedimentation between 20,000 and 780,000 years before the present (YBP) (Zupan Hajna et al., 2008b).

Sample S5 was karst soil collected from beneath the vegetation above the entrance to Pivka jama containing organic soil material, small roots and weathered limestone. Sample S6 was collected in Črna jama, from the bed of a side branch of the Pivka River, approximately 3,000 m from the ponor. Sample S7 was collected in Planinska jama, from the bed of the Pivka River upstream of its confluence with the Rak River (10,000 m from the Pivka River’s ponor). Sample S8, also from Planinska jama, was collected from the bed of the Unica River. Because of its position relative to the large cave entrance, this location is exposed to indirect sunlight (Figure 1). Sampling of cave sediments was approved by the Slovenian Environment Agency, Ministry of the Environment and Spatial Planning, Republic of Slovenia (No. 35602-3/2015-4).

### 2.3 Physical and chemical characteristics of sediments

Physical and chemical analyses of sediments were performed at AGRO-LA, Jindřichův Hradec (Czech Republic) according to ISO methods and national standards of the Czech Republic (ČSN) of the Czech Central Institute for Supervising and Testing in Agriculture (Zbíral, 1995): pH, dry mass, ash content, C, N, P, S, Na, Ca, Mg, K, and heavy metals As, Be, Cd, Co, Cr, Cu, Hg, Ni, Pb, V, and Zn (see Table 1 for individual methods applied).

**Table 1:** Physicochemical analyses of cave sediment samples (S1–S4, S6–S8) and karst soil (S5) from the study, compared with the sediment sample results from the cave Divaška jama (Oarga-Mulec et al., 2021).

Parameter	Unit in DM	Method	Meas. uncertainty	Limit value	S1	S2	S3	S4	S5	S6	S7	S8	Divaška jama
Ash	%	ISO 11465	±5		83.4	86.1	93.9	92.2	85.1	**96.6**	96.3	95.8	96.0
Dry mass	%	ISO 11465	±5		48.0	46.2	64.5	66.4	60.2	57.0	**71.8**	70.1	77.3
pH		ISO 10390	±0.1		6.75	6.81	7.26	7.17	3.95	7.05	7.28	**7.32**	7.20
C	%	ISO 10694	±15		**16.60**	13.90	6.11	7.83	14.90	3.42	3.72	4.22	3.96
N	%	ISO 5663	±15		**1.18**	1.07	1.02	0.68	0.73	0.62	0.56	0.68	0.28
P	%	ISO 11263	±20		**0.91**	0.60	0.50	0.71	0.22	0.78	0.63	0.53	0.46
S	%	ISO 15178	±20		0.01	0.02	0.01	**0.08**	0.02	0.01	0.01	<0.01	0.01
As	mg/kg	ISO 15586	±20		6.7	3.9	8.8	10.1	**10.3**	6.8	7.1	5.4	8.6
Be	mg/kg	ISO 15586	±20		0.83	0.69	1.34	**4.44**	0.79	0.73	0.63	0.63	1.02
Ca	%	ČSN 46 7092	±20		1.74	**4.65**	1.97	0.74	0.32	1.36	1.77	2.57	5.21
Cd	mg/kg	ISO 5961	±20	1	<0.40	<0.40	<0.40	**3.15**	<0.40	<0.40	<0.40	<0.40	<0.40
Co	mg/kg	ISO 8288	±20		**18.4**	13.4	22.5	26.0	7.9	17.4	16.6	13.6	25.3
Cr	mg/kg	ISO 5961	±20	100	87.7	71.4	78.1	**155.0**	53.4	60.4	52.6	41.3	109
Cu	mg/kg	ISO 5961	±20	60	38.0	32.2	**46.8**	41.2	14.3	35.3	26.7	20.6	48.7
Hg	mg/kg	ČSN 75 7440	±15	0.8	0.09	0.07	**0.51**	0.07	0.11	0.11	0.08	0.06	0.08
K	%	ČSN 46 7092	±20		0.47	0.19	**0.61**	0.58	0.10	0.22	0.20	0.16	1.00
Mg	%	ČSN 46 7092	±20		0.59	0.78	0.75	0.59	0.41	0.62	0.61	**0.85**	0.79
Na	%	ČSN 46 7092	±20		0.02	0.02	0.01	**0.03**	0.01	0.01	0.01	0.01	0.02
Ni	mg/kg	ISO 5961	±20	50	79.3	63.7	**91.7**	85.0	30.8	69.2	60.5	48.0	130
Pb	mg/kg	ISO 5961	±20	85	24.7	24.9	19.5	**46.3**	35.4	25.5	20.0	16.4	11.8
V	mg/kg	ISO 15586	±20		55.0	42.1	87.3	**282.0**	87.6	57.0	48.1	48.8	78.3
Zn	mg/kg	ISO 5961	±20	200	**256.0**	84.6	111.0	162.0	59.4	117.0	95.8	79.5	97.9

In bold: highest values; underline: lowest values; DM: dry mass; shaded: exceed the maximum acceptable value for heavy metals in soil (decree on the management of sewage sludge from urban wastewater treatment plants, Uradni list RS 2008, 62/2008); ČSN, National standards of the Czech Republic. The stated measurement uncertainty is the product of the standard measurement uncertainty and the expansion coefficient *k* = 2, which corresponds approximately to 95% coverage probability for a normal distribution.

### 2.4 Microbial cultivation and physiological responses

Four media were used to estimate the cultivable part of the microbial community: nutrient agar (NA, Fluka, Seelze, Germany), Ridacount Total aerobic count (RI, R-Biopharm, Darmstadt, Germany), sediment agar (SA), which contained 1.0% of cave sediment, and 1.5% agar dissolved in distilled water and tap water agar (TA), which contained 1.5% agar dissolved in tap water. Sediment samples (2–4 g) were suspended in 15 mL of 0.9% physiological saline solution, serially diluted (up to 10^−5^), and plated: 0.1 mL per 9-cm-diameter Petri plate and 1 mL per Ridacount plate. Plates were incubated aerobically at 10 °C for 28 days, at 20 °C for 14 days, and at 30 °C for 7 days. A subset of Ridacount plates was cultivated anaerobically using GENbag Anaer airtight bags (BioMérieux, Marcy-l’Étoile, France). Grown colonies were counted, expressed as colony-forming units (CFU) per dry weight (DM), and calculated as the mean concentration value.

Community-level physiological profiling (CLPP) of microbial communities and their physiological responses on 31 organic substrates was observed using Biolog EcoPlates (Hayward, California, United States) (Garland and Mills, 1991). The inoculum was adjusted in 0.9% physiological saline solution to ~150 CFU per well, based on colony counts retrieved on NA at 20 °C. Different cultivation conditions were used to assess the CLPP: temperature (10 °C, 20 °C, 30 °C), period of cultivation (7, 7.5, 13.5, 14, 14.5, and 28 days), and oxygen availability (aerobic, anaerobic) (Table 2). Microwell plates that were cultivated anaerobically were cultivated further aerobically. GENbag Anaer airtight bags were used for the period of anaerobic cultivations. Absorbances at 590 (microbial metabolic activity) and 750 nm (correction for background turbidity) were measured on a microplate reader (Biolog Microstation, BioTek Instruments, Winooski, Vermont, United States) with reading frequencies of 12 h for samples cultivated at 20 °C and 30 °C, and 48 h for samples cultivated at 10 °C. The threshold for positive readings of OD_590_ > 0.400 was set as previously described (Verschuere et al., 1997), and average well color development was calculated and used for analyses. Community metabolic diversity (CMD) and average metabolic response (AMR) were calculated to characterize the microbial community. CMD is calculated by summing the number of positive metabolic responses, and AMR is calculated as the sum of the difference between the OD_590_ of the C-source-containing wells and the control well, divided by the number of C-sources (i.e., 31).

**Table 2:** Cultivation conditions to access CLPP.

Temperature	Oxygen conditions	Cultivation period (h/days)	Oxygen conditions	Cultivation period (h/days)
10 °C	Aerobic	336 h (14 days)	–	–
	Anaerobic*	672 h (28 days)	Aerobic	672 h (28 days)
20 °C	Aerobic	336 h (14 days)	–	–
	Anaerobic*	348 h (14.5 days)	Aerobic	324 h (13.5 days)
30°C	Aerobic	168 h (7 days)	–	–
	Anaerobic*	180 h (7.5 days)	Aerobic	168 h (7 days)

* Continuation with aerobic cultivation.

### 2.5 DNA isolation, amplicon sequencing, bioinformatics analysis, and data availability

High molecular-weight DNA was isolated with a MoBio Power Soil®DNA isolation kit (Qiagen, Hilden, Germany). DNA purity and concentration were determined spectrophotometrically based on standard absorbances at 260 and 280 nm on a NanoDrop Lite Spectrophotometer (Thermo Fischer Scientific, Waltham, Massachusetts, United States). The 260/280 absorbance ratio of the samples ranged from 1.45 to 1.82.

16S rRNA gene amplicon metagenomic analysis was based on 454 pyrosequencing diversity assay (bTEFAP^®^^1^) to assess the bacterial diversity (Dowd et al., 2008; Smith et al., 2010) using barcoded 16S universal eubacterial primers (forward, 27Fmod 5′ AGR GTT TGA TCM TGG CTC AG 3′, reverse, ill519Rmod 5′ GTN TTA CNG CGG CKG CTG 3′). A single-step 30-cycle PCR using HotStarTaq Plus Master Mix Kit (Qiagen, Hilden, Germany) was applied under the following conditions: 94 °C for 3 min, followed by 28 cycles of 94 °C for 30 s, 53 °C for 40 s, and 72 °C for 1 min, with a final elongation at 72 °C for 5 min. Following PCR, all amplicon products from different samples were mixed in equal concentrations and purified using Agencourt Ampure beads (Agencourt Bioscience Corporation, Beverly, Massachusetts, United States). Samples were sequenced utilizing Roche 454 FLX titanium instruments (454 Life Sciences, Brantford, Connecticut, United States).

The Mothur v.1.148.0 software package was used to analyse the generated sequences, and followed the 454 pyrosequencing standard operating procedure (Schloss et al., 2011). Briefly, the sequences were demultiplexed and filtered for quality, dereplicated, and then aligned to the Silva 16S rRNA database v.132. Poorly aligned sequences were filtered, and the error rate was reduced by allowing one mismatch per 100 bp. Chimeras were detected using UCHIME as implemented in mothur. For taxonomic classification, the RDP Naïve Bayesian Classifier was used with an 80% confidence threshold, upon which non-target sequences were removed. OTUs were defined at the genus level (97% sequence similarity in 16S rRNA gene sequence). To account for differences in sequencing depth across samples, the OTU table was normalized by subsampling (rarefaction) to the minimum library size. Alpha diversity was estimated using the Shannon and Simpson indices. The analysis was performed with the R software (R Core Team, 2017) package phyloseq v1.26.1 (McMurdie and Holmes, 2013) and Vegan community ecology v2.5–5 (Oksanen et al., 2019). Vegan community ecology was also used to produce rarefaction curves based on the observed species index, while both vegan and Bioconductor (Reimers and Carey, 2006) were used to produce heatmaps. The results were visualized using ggplot2. The negative control (Fierer et al., 2025) was not considered in data analysis.

Hierarchical clustering for heavy metal data was performed in R. The data were standardized, a distance matrix was computed using the Euclidean distance metric, and hierarchical clustering was performed using the complete linkage method. The resulting object was visualized using ggplot2.

Spearman correlation analysis was performed in R using the rcorr() function from the Hmisc package (Harrell, 2025). Statistically significant associations (*p* < 0.05) were annotated. Clustered heatmaps were generated using the pheatmap package (Kolde, 2025).

## 3 Results and discussion

### 3.1 Sediment characteristics

Physicochemical analyses were conducted to obtain data on habitat characteristics that influence microbiota physiology after being introduced into the karst underground by the river. The concentrations of macronutrients C and N, including the C/N ratio, were the highest in S1 and S2 (alluvial sediments deposited close to the cave entrance) and in the S5 samples (karst soil). High organic content in all of them was already evidenced macroscopically by the presence of leaves and other organic litter material. In the older sediments comprising sample S4, the concentration of C was in the range of that in recently deposited sediments (S3). The concentration of P was the highest in the S1 sample, and the lowest in S5, while the other samples contained comparable concentrations. The concentration of S, which was low in all samples, was highest in S4 (the older sediment). pH was around neutral, except in the karst soil (S5), which was acidic, i.e., 3.95 (Table 1).

The S4 sample contained the highest concentration of metals, specifically Be, Cd, Cr, Na, Pb, and V (Table 1). This cannot be attributed to recent human activities but to heavy minerals that are present naturally. The composition of heavy metals in cave sediments is influenced by the depositional environment (Frierdich et al., 2011). In modern sediments, certain heavy metals can be related to human activity, e.g., Cd in fertilizers (Reimann et al., 2012). Some heavy metals are concentrated during weathering processes of Earth materials, for example, during bauxitization (Mordberg, 1993), laterization (Butt and Cluzel, 2013), and kaolinization (Dill, 2016). Most of the sediments in the PPCS originate from flysch successions, specifically from different stratigraphical horizons within them, which can contribute different heavy mineral content and assemblage composition related to erosional exposure of different parent lithologies in the source region (Zupan Hajna et al., 2008a, 2020). In similar fashion to S4, other older alluvial sediment sampled in a different karst cave (Divaška jama, SW Slovenia), which was deposited during a similar timeframe—from pre-0.97 Ma to 0.73 Ma (Bosák et al., 1998)—as the S4 sediment in Postojnska jama, exhibited comparable concentrations of some heavy metals (Oarga-Mulec et al., 2021).

Cd was present only in the older sediment (S4). With the exceptions of As and Hg, which displayed their highest concentrations in the karst soil (S5), concentrations of other metals were lower in this sample than in the cave sediments (Table 1). The nature of soil formation processes in karst areas is specialized, because various major compounds that are weathered from minerals containing, for example, K, Na, Ca, and Mg, which are present in the carbonate rocks and suffer dissolution and leaching by water. During the initial phase of karst soil formation, a slightly alkaline environment prevails, but conditions later become more acidic, thus facilitating the leaching of heavy metals. In karst soils, heavy metals are commonly quite stable; only a small proportion is leached into the environment, within the pH range from 6 to 8. In the case of an acidic pH, this proportion is far higher, e.g., at pH 3, it is 4.04% for Cd, 2.43% for Ni, and 1.69% for Zn (Ji et al., 2021).

**Figure 2:**
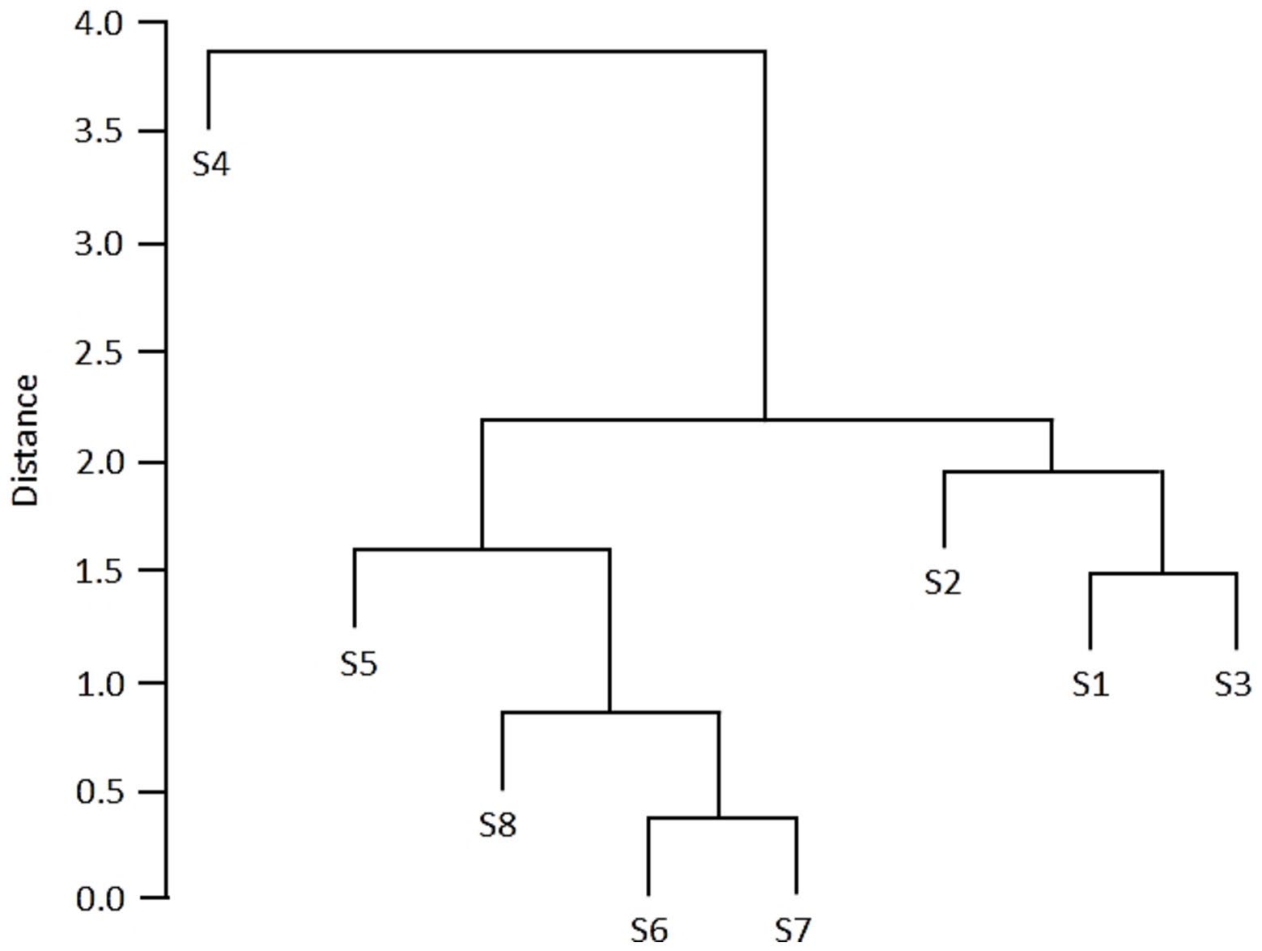
Hierarchical clustering dendrogram of samples S1–S8 based on physicochemical parameters (Table 1).

Hg and Ni were present in all samples, with the highest concentration in the S3 sample, which was collected from the site that is flooded by the Pivka River during high-water periods. The highest concentration of Zn, which was measured in sample S1, exceeded the maximum acceptable value for this heavy metal in soil. Regarding the legislation, the maximum acceptable values for heavy metals in soil were also exceeded for Ni (S1, S2, S3, S4, S6, S7), Cr (S4), and Cd (S4) (Table 1). A hierarchical clustering dendrogram showed two main clusters: one composed of samples S5, S6, S7, and S8, and another of S1, S2, and S3, whereas S4 is a clear outlier because of its high dissimilarity. Within the clusters, S6 and S7, as well as S1 and S3, are the most closely related pairs (Figure 2).

### 3.2 Microbiota in sediments

Cultivation of microorganisms from sediments was conducted to screen for their viability, concentration, and to prepare a uniform inoculum in terms of concentration for the CLPP experiment to observe quantitative metabolic responses of microbiota after supplementing with organic compounds. Microorganisms from sediments were retrieved from all samples on nutrient-poor and nutrient-rich media. The S4 sample exhibited the lowest concentration of cultivable cell count. No colonies were detected on Ridacount plates when this sample was cultivated anaerobically. The S8 sample (sediment from the Planinska jama cave entrance) exhibited the highest microbial biomass on most of the media employed (10^5^–10^6^ CFU/ g DM) (Table 3).

**Table 3:** Cultivable microorganisms in different growth conditions in sediment samples.

Medium	Oxygen	Temp. (°C)	Cultivation period (days)	×10^4^ CFU/g DM							
				S1	S2	S3	S4	S5	S6	S7	S8
NA	Aerobic	10	28	**68 (43)**	4 (4)	62 (45)	5 (4)	8 (8)	35 (6)	43 (25)	55 (36)
	Aerobic	20	14	113 (64)	46 (32)	156 (42)	12 (10)	32 (7)	42 (1)	82 (36)	**641 (605)**
	Aerobic	30	7	24 (21)	14 (14)	45 (34)	0.5 (0.7)	7 (7)	31 (0)	102 (62)	**218 (127)**
SA	Aerobic	10	28	51 (0)	24 (2)	60 (62)	12 (6)	89 (26)	28 (21)	41 (24)	**160 (100)**
	Aerobic	20	14	**511 (660)**	88 (88)	184 (21)	20 (14)	109 (13)	101 (80)	93 (8)	252 (88)
	Aerobic	30	7	50 (6)	47 (88)	**132 (51)**	5 (4)	33 (13)	75 (21)	24 (24)	91 (17)
TA	Aerobic	10	28	37 (8)	10 (7)	109 (15)	3 (2)	4 (5)	57 (5)	**122 (66)**	56 (40)
	Aerobic	20	14	73 (38)	89 (29)	**228 (103)**	12 (9)	45 (12)	192 (159)	76 (8)	74 (55)
	Aerobic	30	7	15 (11)	258 (223)	61 (51)	0.6 (0.8)	**278 (86)**	26 (51)	53 (49)	146 (56)
RI	Aerobic	10	28	417 (107)	164 (69)	434 (73)	6 (3)	112 (59)	147 (38)	226 (160)	**524 (182)**
	Aerobic	20	14	425 (44)	144 (27)	338 (243)	6 (4)	57 (41)	330 (206)	520 (232)	**559 (248)**
	Aerobic	30	7	239 (20)	76 (54)	381 (43)	10 (2)	46 (34)	268 (93)	477 (135)	**762 (342)**
RI	Anaerobic	10	28	6 (8)	3 (4)	8 (7)	0 (0)	1 (1)	13 (9)	27 (21)	**34 (3)**
	Anaerobic	20	14	31 (27)	13 (11)	18 (14)	0 (0)	2 (3)	44 (31)	**119 (41)**	97 (84)
	Anaerobic	30	7	16 (22)	18 (13)	25 (22)	0 (0)	3 (5)	95 (28)	**131 (283)**	126 (43)

Data are mean (standard deviation); in bold: highest values; underline: lowest values; DM, dry mass; NA, Nutrient agar; SA, Sediment agar; TA, Tap water agar; RI, Ridacount Total aerobic count.

16S rRNA gene amplicon metagenomic analysis was conducted to detail the taxonomic composition of Bacteria. The total count of high-quality sequences was 57,770, equivalent to 5,566 ± 1936.4 sequences (mean ± SD) per sample (Table 4). Rarefaction curves depicting sampling effort are presented in Supplementary Figure S2. From these curves, it is evident that, apart from samples S3 and S4, additional sampling would have yielded evidence of additional diversity.

**Table 4:** Number of reads, OTUs, and Shannon and Simpson indices.

Diversity metrics	S1	S2	S3	S4	S5	S6	S7	S8
Number of bacterial sequences	10,662	8,296	4,784	2,553	8,328	7,510	9,125	6,512
Number of bacterial genus-level OTUs	217	228	137	55	135	218	179	214
Shannon index	2.37	2.47	2.07	1.97	1.91	2.52	2.45	2.36
Simpson index	0.86	0.89	0.81	0.79	0.82	0.90	0.89	0.86

Total genus-level OTU numbers, Shannon’s, and Simpson’s indices were used to evaluate alpha-diversity of the samples. The number of OTUs varied from 55 in sample S4 to 227 in sample S2. Shannon’s and Simpson’s indices showed that samples S4 (old alluvial sediment) and S5 (karst soil) were the least diverse. Whereas samples S1, S2, S5, and S6 displayed comparable diversity, the diversity of sample S3 (influenced by the seasonal local flooding of the Pivka River) was comparatively lower.

**Figure 3:**
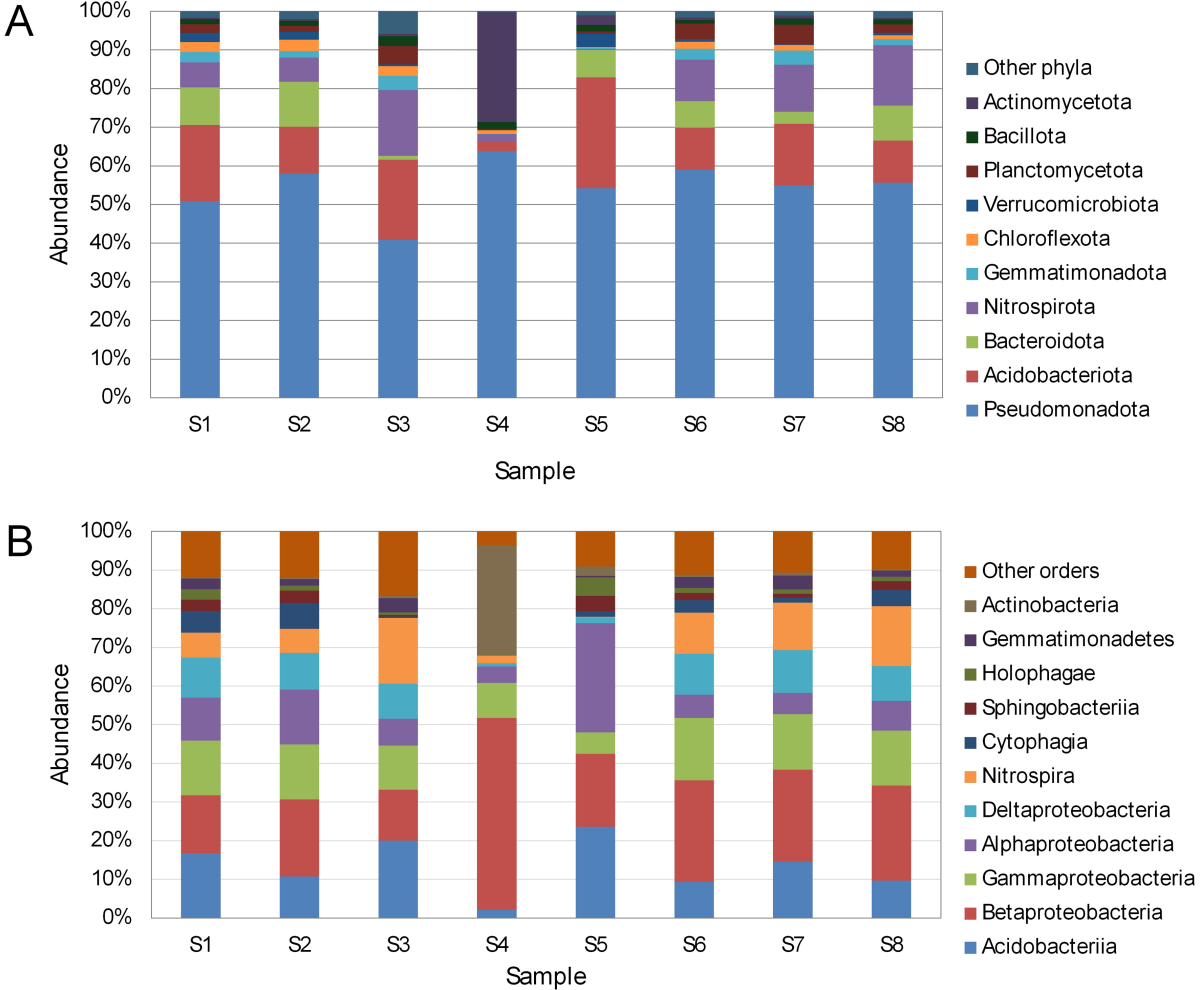
Relative abundances of bacterial phyla **(A)** and classes **(B)**.

Members of 26 bacterial phyla were detected in the samples; the ten phyla most common in sequenced samples are presented in Figure 3A. Pseudomonadota dominated all cave sediment samples with abundances ranging from 40.9% in S3 to 63.9% in S4. It was followed in abundance by Acidobacteriota (2.4% in S4 to 20.7% in S3) and Nitrospirota (0.2% in S5 to 17.0% in S3). Bacteroidota also represented a significant portion of diversity, with abundances ranging from 0.0% in S4 to 9.8% in S1. Most samples (S1, S2, S5–S8) showed a similar community structure. However, Actinomycetota represented the second most abundant group (28.4%) in sample S4, whereas its abundances ranged between 0.4–2.5% in the remaining samples. In contrast, sample S3, influenced by seasonal flooding of the Pivka River at the micro-location, and temporary stagnant water, contained a higher proportion of Nitrospirota (17.0%), which corresponded with a reduced proportion of Pseudomonadota (40.9%).

At class level (Figure 3B), Acidobacteriia (2.1% in S4 to 23.6% in S5) and Betaproteobacteria (13.1% in S3 to 49.6% in S4) were the most abundant. In terms of community structure, sample S4 stood out because of an abundance of Actinobacteria (28.4%), which represents a minor portion of diversity in other samples (0.4% in S1 to 2.5% in S5). This sample also showed the highest recorded proportion of Betaproteobacteria (49.6%). Sample S3 included the highest recorded proportion of *Nitrospira* (17.0%), whereas sample S5 (karst soil) contained the highest recorded proportion of Alphaproteobacteria (28.2%).

**Figure 4:**
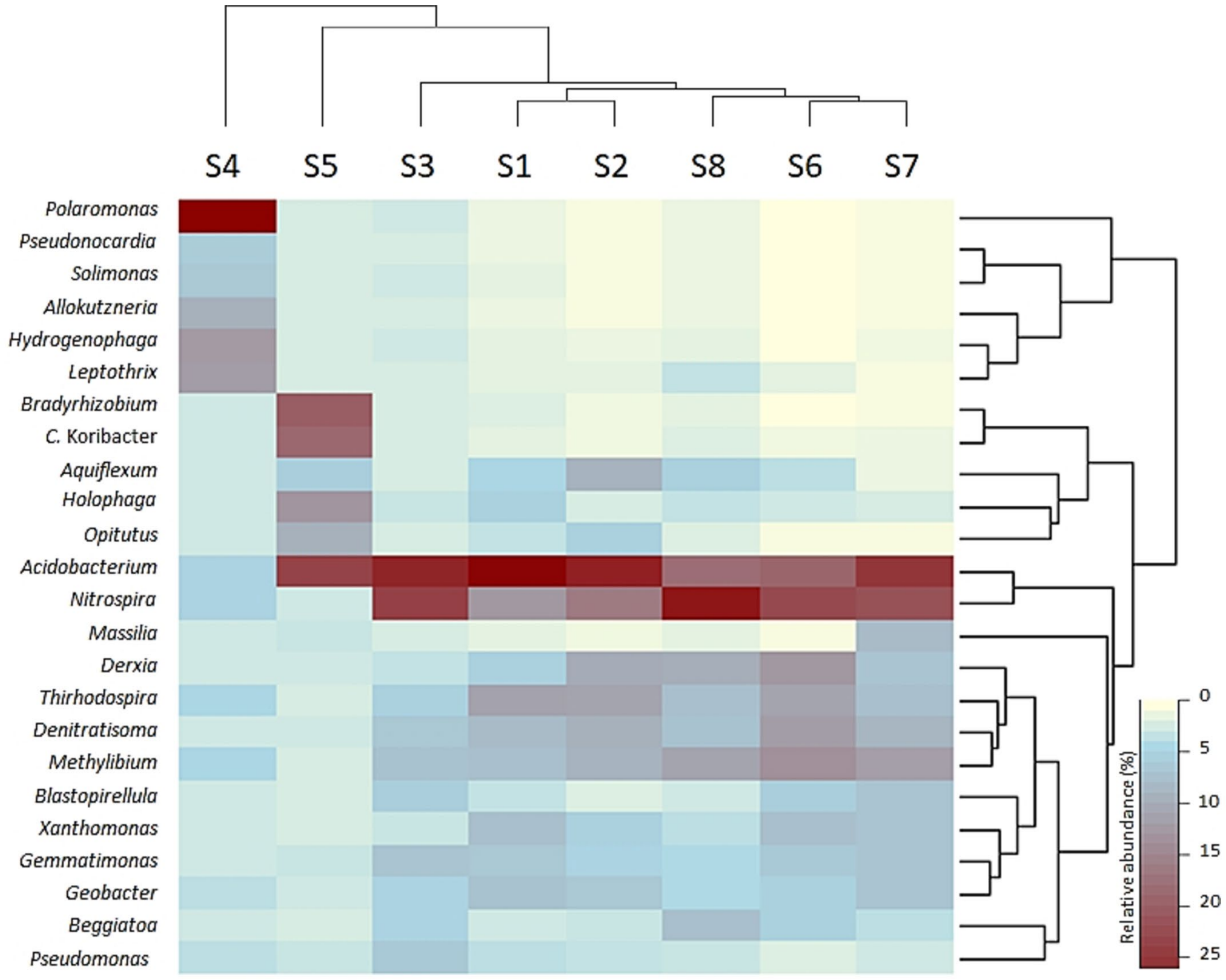
Heatmap and cluster analysis dendrograms showing the 16S rRNA sequence abundances of bacterial genera with abundances over 3% in at least one sample.

A total of 350 genera were detected in the sequenced samples (Supplementary Table S1). A hierarchical clustering cladogram of samples in Figure 4 was similar to the cladogram depicted in Figure 2. Samples S1 and S2 (alluvial sediments deposited close to the cave entrance), as well as S6–S8 (alluvial sediments downstream), formed two distinct groups. Such clustering of sediments reflects the predominant seasonal conditions in the underground Pivka River, when chemical and bacterial parameters display a monotonous trend of decreasing concentrations from the ponor toward the interior of the PPCS during stable hydrological conditions (Mulec et al., 2019). Sample S3 (sediment occasionally covered by floods) branched separately from samples S1 and S2. In terms of dissimilarity, sample S4 (older sediment) branched separately from the other sediment samples and from the control sample S5 (karst soil).

Among the abundant genera, members of *Acidobacterium*, *Denitratisoma, Gemmatimonas*, *Geobacter*, *Holophaga*, *Methylibium*, *Nitrospira*, and *Pseudomonas* were found in all samples and represented a core of these communities. *Acidobacterium* (2.2–16.6%) and *Nitrospira* (0.2–17.0%) were the most abundant genera in all samples except samples S4 and S5. Sample S4 was dominated strongly by members of *Polaromonas* (26.2%), a typical soil psychrophile (Du et al., 2025). Whereas *Acidobacterium* was abundant in sample S5, *Nitrospira* represented only a minor component of the community (0.2%).

Several bacterial genera involved in nitrogen cycling were abundant. Apart from *Nitrospira,* as mentioned above, which is involved in nitrite oxidation (Daims and Wagner, 2018), nitrate-reducing *Denitratisoma* (Fahrbach et al., 2006) were identified, as well as nitrogen-fixing *Derxia* (Xie and Yokota, 2004) and *Bradyrhizobium* (Rivas et al., 2009). A chemolithotrophic sulfur-oxidizing bacterium, *Beggiatoa* (Teske and Salman, 2014), represented more than 0.5% of the community found in samples S1–S3 and samples S6–S8. *Methylibium*, a methylotroph, was found in all samples at abundances greater than 2% except in the karst soil sample (S5), where its abundance was 0.05%. *Hydrogenophaga* (H_2_-oxidizer, chemolithoautotroph, chemoorganotroph) was abundant in sample S4 (8.5%). Other chemolithotrophic bacteria included *Thiorhodospira* (sulfide oxidation), *Thiobacter* (ammonia oxidation), and *Nitrosovibrio*. However, most of the community comprised chemoorganotrophs, some with versatile metabolism. Some examples include *Steroidobacter,* which degrades complex organic molecules, including steroids (Fahrbach et al., 2008), complex polymer-degrading *Massilia* and *Ohtaekwangia* (Amirhosseini et al., 2025; Babu et al., 2019), and pollutant-degrading *Polaromonas* (Mattes et al., 2008).

Heavy metals act as selective pressures that influence and shift microbial community structure and disrupt ecosystem functions (Xiao et al., 2025). Several taxa, such as *Acinetobacter*, *Bacillus*, *Halomonas*, *Lactobacillus*, *Marinobacter*, *Paracoccus*, *Pseudomonas*, *Streptococcus*, *Sulfobacillus*, and *Sulfurifustis* tolerate heavy metal stress well. Functional predictions revealed that these taxa can employ various survival strategies, including extracellular polymerization, nutrient uptake, intracellular sequestration, and active efflux systems (Zhao et al., 2025). The detection of elevated heavy-metal concentrations within the older cave-sediment layer (sample S4) can be correlated with taxa that carry metal-resistance genes. For example, Pseudomonadota and Actinomycetota were dominant in this sample, implying long-term selection for metal-tolerant microbial groups. Metal-resistance genes (MRGs) to Pb, Ni, Hg, W, Zn, Ag, Cr, Fe, As, Cu, along with antibiotic resistance genes (ARG), have previously been identified within lake sediments, with Pseudomonadota, Euryarchaeota, Actinomycetota, Chloroflexota, and Bacteroidota as potential hosts (Du et al., 2022). Enhanced extracellular polymeric substance production and biofilm formation might provide protection for microorganisms under combined metal and flood stress, consistent with cave survival strategies countering oligotrophy (Ghezzi et al., 2022), heavy-metal exposure (Burow et al., 2019; Hamedi et al., 2019), and episodic hydrological disturbances (Martin-Pozas et al., 2024).

### 3.3 Correlations between the environmental parameters and major members of bacterial communities

**Figure 5:**
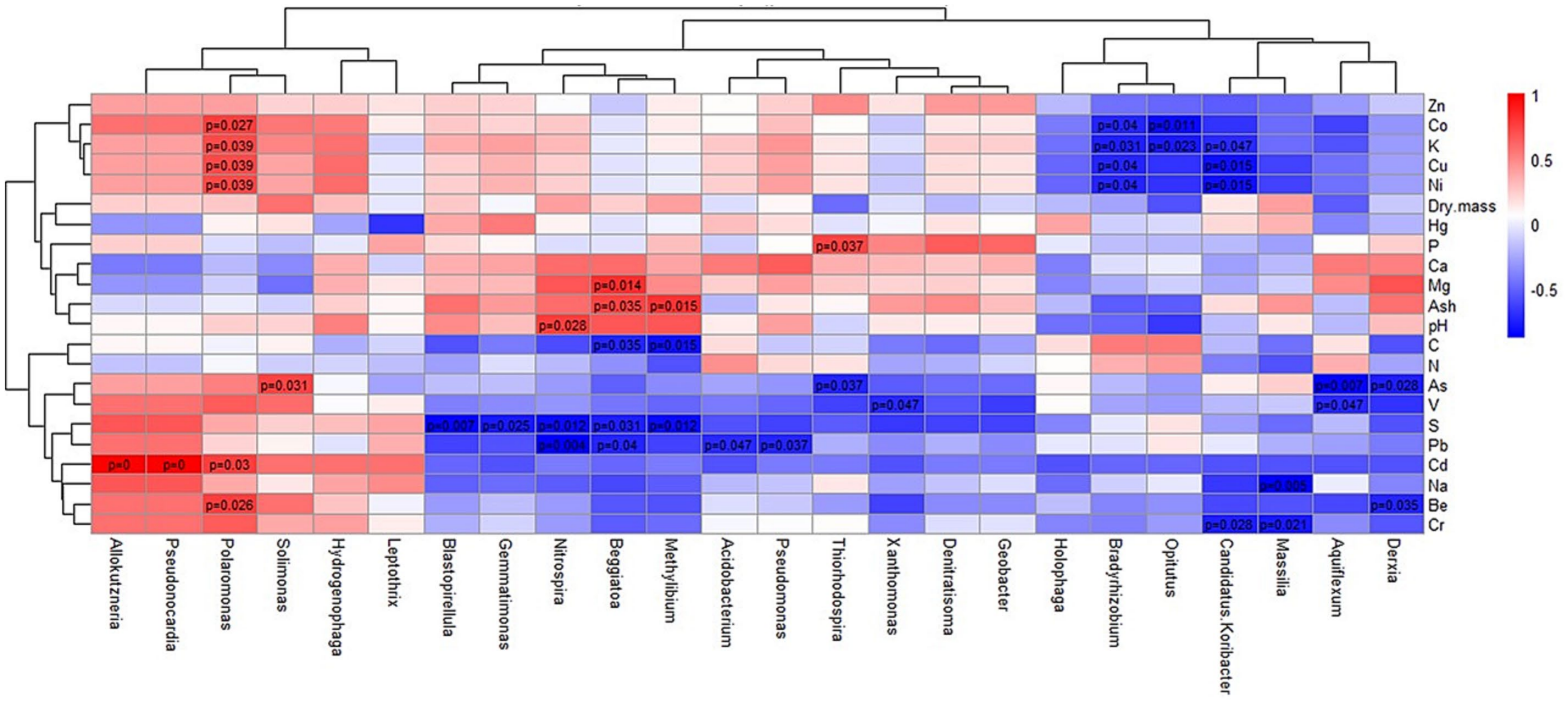
Heatmap and hierarchical cluster dendrogram showing Spearman correlation coefficients between bacterial genera (with relative abundances exceeding 3% in at least one sample) and measured environmental parameters. Statistically significant correlations (*p* < 0.05) are indicated by their corresponding *p*-values.

Spearman correlation analysis revealed distinct and statistically significant associations (*p* < 0.05) between bacterial taxa and environmental parameters, including heavy metal concentrations. The resulting heatmap (Figure 5) showed three major clusters of bacterial taxa, whose composition corresponded to their ecological origins (see Figure 4 for comparison): (1) highly resistant taxa included genera predominant in old sediment (sample S4), which generally had high heavy metal concentrations and, among all samples, the highest levels of Be, Cd, Cr, Pb, and V; (2) moderately sensitive taxa found in recently deposited sediments (samples S1–S3 and S6–S8); and (3) highly sensitive taxa found in the soil sample collected above the cave Pivka jama (sample S5).

The first cluster comprised *Allokutzneria*, *Pseudonocardia*, *Polaromonas*, *Solimonas*, and *Hydrogenophaga*, which were positively correlated with heavy metals. *Allokutzneria* and *Pseudonocardia* showed strong and significant positive correlations with Cd, while *Solimonas* showed a significant positive correlation with As, suggesting active adaptation to metal-rich conditions. *Polaromonas*, one of the predominant genera in sample S4, showed strong positive correlations with several heavy metals (i.e., Be, Cd, Ni, Cu, and Co). Members of this genus are known to carry plasmids that confer this type of resistance (Ciok et al., 2018). The taxa in this group represent a metal-resistant assemblage adapted to the elevated concentrations typically found in long-term contaminated sediments, such as sample S4.

The second cluster comprised taxa predominantly associated with recently deposited sediments (samples S1–S3 and S6–S8), where they represented either dominant community members (*Acidobacterium*, *Nitrospira*) or moderately abundant taxa, including *Blastopirellula*, *Gemmatimonas*, *Beggiatoa*, *Methylibium*, *Pseudomonas*, *Thiorhodospira*, *Xanthomonas*, *Denitratisoma*, and *Geobacter*. This cluster showed a mixture of positive and negative correlations with environmental parameters. Significant negative correlations with heavy metal concentrations were detected, notably between Pb and *Nitrospira*, *Beggiatoa*, *Acidobacterium*, and *Pseudomonas,* as well as between *Thiorhodospira* and As, and *Xanthomonas* and V. In addition, five genera – *Blastopirellula*, *Gemmatimonas*, *Nitrospira*, *Beggiatoa*, and *Methylibium –* showed significant negative correlations with sulfur. Among these, *Nitrospira*, *Beggiatoa*, and *Methylibium* displayed complex response patterns, exhibiting both positive and negative correlations with other parameters such as Mg, C, ash content, and pH.

The third cluster comprised the genera *Holophaga*, *Bradyrhizobium*, *Opitutus*, *Candidatus Koribacter*, *Massilia*, *Aquiflexum*, and *Derxia*, which represented a substantial portion of the microbial diversity in the soil sample collected above the cave Pivka jama (sample S5). These taxa were sensitive to heavy metals and various other measured compounds and environmental parameters, showing predominantly negative correlations. Notable negative correlations were observed for *Bradyrhizobium*, *Opitutus*, and *Candidatus Koribacter* with Co, Cu, and Ni; *Candidatus Koribacter* and *Massilia* with Cr; *Aquiflexum* with As and V; and *Derxia* with As and Be. Additionally, some taxa showed significant negative correlations with other compounds, particularly *Opitutus* with K and *Massilia* with Na.

### 3.4 Microbial metabolism

**Figure 6:**
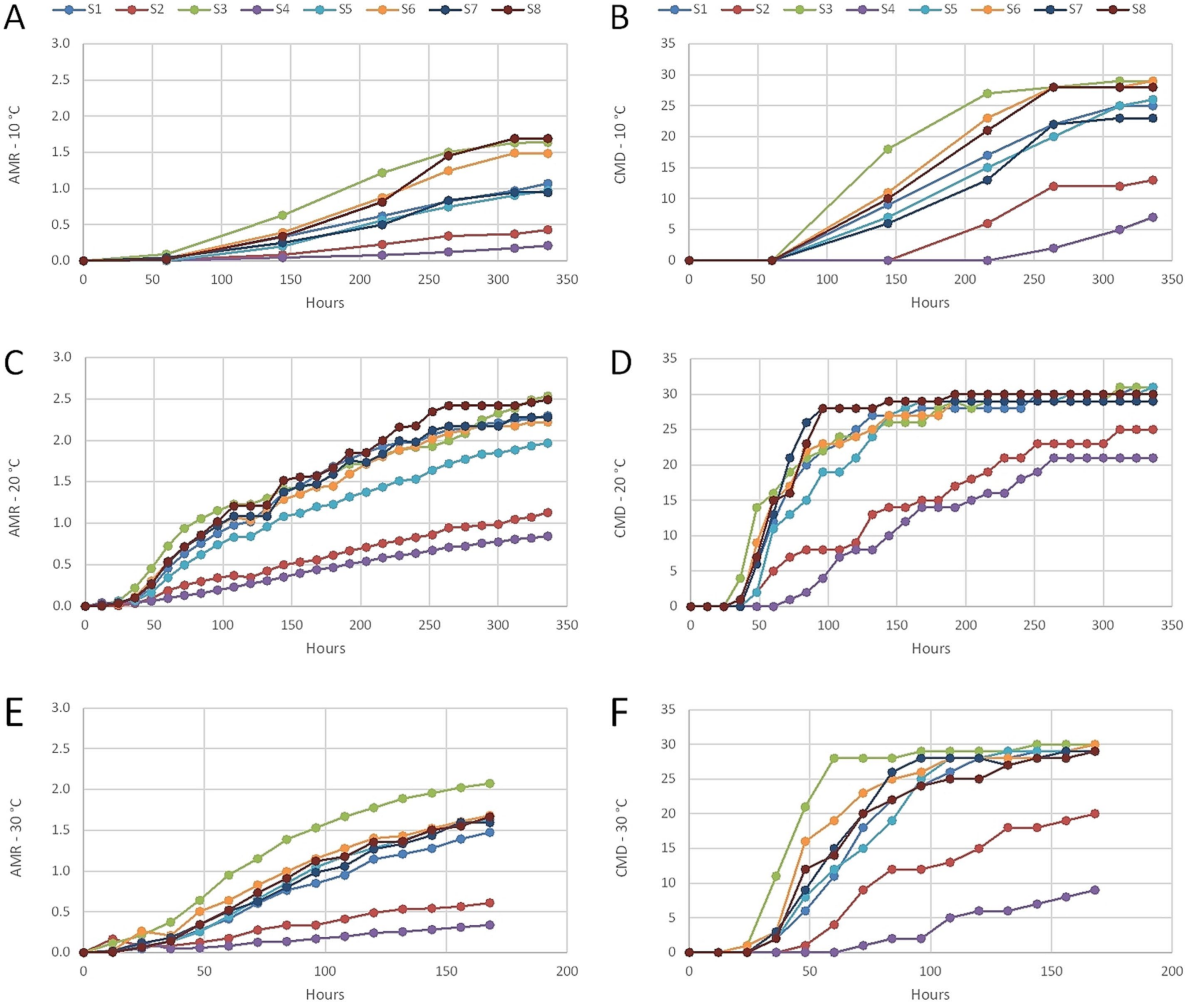
CLPP profiles of samples comparing the AMR (left) and CMD (right) in time. **(A,C,E)** AMR during aerobic cultivation at different temperatures (10 °C, 20 °C, 30 °C). **(B,D,F)** CMD during aerobic cultivation at different temperatures (10 °C, 20 °C, 30 °C).

The CLPP of microbiota from cave sediments was evaluated along with both the average metabolic response (AMR)—which describes the average respiration of the C-sources by the microbial community—and the community metabolic diversity (CMD), which is determined by the number of substrates utilized by the microbial community and is analogous to community functional richness. All 31 organic compounds were metabolized aerobically at 20 °C in samples S1 (recently deposited sediment close to the cave entrance), S3 (sediment from a side branch of the Pivka River that is sporadically flooded), and S5 (karst soil). The lowest AMR and CMD at all three test temperatures were for S4 (old sediment) and S2 (recently deposited alluvial sediment close to the cave entrance, Figure 6). During aerobic cultivation at 10 °C, the highest AMR was for S3 (1.64) and S8 (1.62), and the highest CMD was for S3 and S6 (both 93.5%). Cultivation at 20 °C reflected in the highest AMR for S3 (2.53) and S8 (2.49), and the highest CMD was for S1, S3, and S5 (all 100.0%). During aerobic cultivation at 30 °C, the highest AMR was for S3 (2.07), and the highest CMD was for S3 (96.8%) and S6 (96.8%). During aerobic cultivation the AMR was the highest at 20 °C ranging from 0.85 to 2.53, followed by AMR at 30 °C ranging from 0.61 to 1.68 and finally by AMR at 10 °C ranging from 0.21 to 1.64. At these oxygen conditions the CMD was the highest at 20 °C (67.7–100.0%), next at 30 °C (29.0–96.8%) and finally at 10 °C (22.6–93.5%). During anaerobic cultivation the AMR was highest at 20 °C, ranging from 0.05 to 0.56, followed by the AMR at 10 °C, ranging from 0.05 to 0.41. Finally, the AMR at 30 °C ranged from 0.07 to 0.12. Under these conditions, the CMD was highest at 20 °C (0.0–54.8%), next at 10 °C (0.0–35.5%), and finally at 30 °C (0.0–9.7%). During anaerobic–aerobic cultivation the AMR was the highest at 30 °C, ranging from 0.56 to 1.38, followed by the AMR at 20 °C, ranging from 0.50 to 1.13. Finally, the AMR at 10 °C ranged from 0.66 to 1.08. Under these conditions the CMD was the highest at 30 °C (48.4–93.5%), next at 20 °C (45.2–80.6%), and finally at 10 °C (41.9–74.2%) (Supplementary Tables S2–S4; Supplementary Figure S3).

There were differences regarding catabolism of the tested compounds. Under aerobic cultivation, less than 50% of the samples metabolized substrates: B3 (D-galacturonic acid), C2 (i-erythritol), E3 (γ-hydroxybutyric acid), F3 (itaconic acid), and H1 (α-D-lactose) at 10 °C; E3 and F3 at 20 °C; and only E3 and F3 at 30 °C. If samples were cultivated first anaerobically and followed then by aerobic conditions, there were more compounds not metabolized in less than 50% of cave samples. This accounts for compounds at 10 °C for B1 (pyruvic acid methyl ester), B2 (D-xylose), B3, B4 (L-asparagine), C1 (Tween 40), C2, C3 (2-hydroxy benzoic acid), D3 (4-hydroxy benzoic acid), D4 (L-serine), E3, F1 (glycogen), F3, G3 (α-ketobutyric acid), at 20 °C for B1, B2, C1, C3, D3, D4, E3, F1, F3, G3, at 30 °C for B3, C1, E3, F1, F3 (Supplementary Table S5). The least favorable compounds for utilization by cave microorganisms were γ-hydroxybutyric acid (E3), which occurs naturally in almost all living species (Doherty et al., 1978; Roth and Giarman, 1970), and itaconic acid (F3), which is commonly produced by fungi and used in many industrial applications (Hevekerl et al., 2014; Kawamura et al., 1981).

Sediment transported into the PPCS by the Pivka River contains different material originating from geological (e.g., erosion), and biological processes including agricultural runoff and treated wastewater (Mulec et al., 2019). Many organic compounds are dissolved in the water (Mulec et al., 2019), which is a substrate for microbial metabolism. The sediments contain high concentration of microorganisms. Previous interactions of substrate/pollutant with cave sediment are reflected in the degradation rate of such compounds, as has been shown already in the case of atrazine breakdown in karst groundwater that surface-derived catabolic genes allowed microbial communities to degrade atrazine rapidly (Iker et al., 2010). Flooding promotes physical contact, drift of microorganisms between sites and environmental changes. Underground sites that face only sporadic floods and no significant turbulent flow (e.g., sample S3) support a higher metabolic potential for microbial degradation of organic compounds. At such places, there is time for slow settlement of the newly introduced material and its interaction with previously deposited material. Low temperature and, particularly, anaerobiosis reduced the microbial catabolism of the substrates tested. The ability of microbe metabolism to respond rapidly is especially important in microhabitats where changes occur rapidly, such as when flood pulses introduce oxygenated water that contains nutrients. This is particularly relevant for those underground karst habitats that face increased hydrological volatility related to weather and climate changes. Temperature shifts and oxygen pulses during and following floods can either suppress or stimulate microbial processes. Therefore, potentially, sediments that can sustain metabolic function or support its recovery under such fluctuating conditions can play a critical ecological role.

The functional profiles of cave sediments revealed clear differences in metabolic capacity, with the highest diversity observed at sites influenced by the effects of periodic flooding. The sediment from site S3 demonstrated strong microbial adaptability, indicating that hydrological disturbance promotes metabolic versatility. The presence of functionally diverse taxa involved in nitrogen, sulfur, and carbon cycling underscores their role in maintaining biogeochemical processes under fluctuating oxygen and nutrient regimes. Such resilience is particularly relevant under certain future climate scenarios, where increased hydrological extremes are expected to challenge subsurface ecological balances. In this context, these microbial communities might act as ecological buffers, stabilizing essential processes despite environmental variability.

### 3.5 Ecological interpretations

Effects of microbial metabolic capacity, environmental history, and potential for application were examined, to help interpret these results in broader ecological and functional contexts. Cave sediments host microbial communities with functional traits that can be critical, ecologically, for the uptake, degradation and transformation of nutrients and the attenuation and removal of pollutants. The dominance of Pseudomonadota, Actinomycetota and Acidobacteriota reflects communities capable of both aerobic and anaerobic processes, important for nitrogen, sulfur and carbon cycling in subterranean environments. The presence of denitrifiers, e.g., *Denitratisoma*, and chemolithotrophs, e.g., *Beggiatoa*, *Nitrospira*, demonstrates the role of cave sediments in maintaining redox balance under fluctuating hydrological conditions.

This comparative approach, which includes samples of an ancient palaeo-river sediment and of recent alluvial deposits, demonstrates how sediment age and hydrological activity shape microbial community structure and function. Sample S4, deposited hundreds of thousands of years ago, revealed a microbial community with low diversity and restricted metabolism, likely a result of long-term isolation and limited resource input. This suggests that the age of sediments might act as a geochemical filter, with both the accumulation of heavy metals and a reduction of organic input limiting the microbial diversity and the metabolic flexibility. In contrast, the sample S3, from a periodically flooded location, exhibited evidence both of taxonomic richness and of high metabolic potential. These results show the dual value of cave sediments, as microbial “time capsules” that preserve historical community signatures, and as zones of dynamic microbial activity influenced by contemporary hydrological events.

The application of CLPP (quantified via AMR and CMD indices) from this study provides a link between microbial community composition and functional metabolic potential. This approach offers an ecological lens that enables a detailed focus into microbial adaptability and resilience within karst systems under environmental stress, and contributes to gaining a better understanding of subterranean biogeochemical cycling. The temperature sensitivity of AMR and CMD across all oxygen regimes accentuates the importance of thermal stability for microbial functioning in karst sediments. While cave temperatures are typically stable and relatively low (8–12 °C), climate-induced shifts, including warmer winters or anthropogenic heating, may change and reduce microbial metabolic rates. The study results show that activity peaked at 20 °C, with reduced function under both colder (10 °C) and warmer (30 °C) conditions, suggesting narrow mesophilic optima for organic matter turnover. These findings raise concern for subsurface carbon and nitrogen cycling under changing thermal conditions. Moreover, the inclusion of anaerobic–aerobic transitions simulates conditions during post-flood oxygenation pulses, a key recovery phase encountered within cave environments. Increases in substrate utilization observed under these conditions suggest that microbial communities can recover part of their metabolic capacity following oxygen reintroduction, which might be critical for contaminant degradation and organic matter transformation during increasingly frequent hydrological disturbances. The observed ability of cave microbiota to metabolize a wide range of organic substrates under low-oxygen and low-temperature conditions suggests that these communities retain functional potential even in energy-limited environments. Genera such as *Polaromonas* and *Methylibium*, known for degrading hydrocarbons and xenobiotics, might be relevant for the advance of natural attenuation processes within contaminated karst systems.

The periodically flooded sediments (such as the S3 sample) might function as dynamic microbial hotspots, contributing disproportionately to organic matter turnover and pollutant degradation during hydrological events. In contrast, metabolically limited zones (as typified by the S4 sample) might serve as long-term contaminant reservoirs. Such spatial and functional heterogeneity can be integrated further into predictive karst biogeochemical models and might help to forecast carbon and nitrogen fluxes and could also help to guide both targeted monitoring and the management of vulnerable subterranean environments.

## 4 Conclusion

This study demonstrates the value of integrating community-level physiological profiling with microbial taxonomic analysis and the chemical characteristics of the environment to assess ecosystem functioning in karst cave sediments. γ-Hydroxybutyric acid and itaconic acid were the least favorable substrates for cave microorganisms. AMR peaked at 20 °C under both aerobic and anaerobic conditions, with different responses at 10 °C and 30 °C. The lowest AMR and CMD values occurred in older rather than modern sediment.

Sediment age, oxygen availability, temperature, and flooding regime were found to influence community structure and function. The older sediment, which was characterized by the lowest number of OTUs and culturable microbial biomass along with elevated concentrations of Cd, Ni, and Cr, exhibited the lowest diversity and metabolic activity, showing the role of sediment age as a geochemical filter limiting present-day microbial flexibility. In contrast, the sediments from periodically flooded sample sites hosted a distinct, metabolically versatile community dominated by Pseudomonadota. Recognition of functionally relevant taxa such as *Acidobacterium*, *Nitrospira*, *Geobacter*, *Methylibium,* and *Polaromonas* links their presence to key roles in nitrogen, sulfur, and carbon cycling.

Given projected increases in precipitation extremes and contaminant mobilization under weather and climate changes, the observed microbial adaptations in karst sediments can serve as bioindicators of ecosystem resilience.

## Data Availability

The datasets presented in this study can be found in online repositories. The names of the repository/repositories and accession number(s) can be found at: https://www.ncbi.nlm.nih.gov/, PRJNA1275629.

## Footnotes

1
http://www.mrdnalab.com
