# Supplementary material for: Metabolic traits of sediment bacteria in karst caves in the light of environmental changes
Source: Front Microbiol. 2025 Dec 12;16:1724116. doi: 10.3389/fmicb.2025.1724116 (PMC12742472; doi:10.3389/fmicb.2025.1724116)
Supplement: Supplementary file 1 [file Table_1.PDF]

Supplementary table 1: List of genera detected, and details of their relative abundance in individual samples.

Sample	S1	S2	S3	S4	S5	S6	S7	S8
Acidimicrobium	0	0	0	0	0	0.020509	0	0.024728
Acidipila	0	0	0	0	0.553032	0	0	0
Acidisphaera	0.014545	0.05735	0	0	0.908552	0	0	0.098912
Aciditerrimonas	0.043636	0.019117	0	3.157895	0	0.041017	0	0
Acidobacterium	16.56727	10.62894	19.99413	2.155388	9.22378	8.736669	14.2244	9.248269
Acidovorax	0.014545	0.095584	0	0	0	0.041017	0.015328	0
Acinetobacter	0	0	0.05872	1.403509	0	0	0	0
Actinoallomurus	0	0	0	0	0.375272	0	0	0.024728
Actinokineospora	0	0	0	2.506266	0	0	0	0
Actinomadura	0	0	0.05872	0	0	0.123052	0	0
Adhaeribacter	0	0	0	0	0	0.020509	0	0.12364
Aeromonas	0	0	0	0.200501	0	0	0	0.024728
Afipia	0.014545	0.076467	0	0	0.217262	0.020509	0	0.074184
Agrobacterium	0.014545	0.038234	0	0	0	0	0	0
Algibacter	0.014545	0.019117	0	0	0	0.041017	0.015328	0.049456
Algisphaera	0.014545	0	0	0	0	0.041017	0.015328	0
Alkalilimnicola	0	0	0.1468	0	0	0	0	0
Alloactinosynnema	0	0	0	2.005013	0	0	0	0
Allokutzneria	0	0	0	5.964912	0	0	0	0
Alsobacter	0.043636	0.076467	0	0	0	0	0.015328	0.024728
Alteromonas	0	0.095584	0	0	0	0	0	0
Anabaena	0.058182	0	0	0	0	0	0	0
Anaerolinea	0.378182	0.152934	0.910159	0	0	0	0.352544	0.024728
Anaeromyxobacter	1.949091	1.414643	0.968878	0	0.414774	1.312551	1.287554	1.088032
Aquabacterium	0	0.210285	0	0	0	0	0	0
Aquiflexum	1.745455	3.249857	0.11744	0	1.422082	1.579163	0.352544	2.126607
Arcicella	0.887273	0.649971	0	0	0	0.225595	0.521153	0.593472
Arenimonas	0.043636	0.05735	0	0	0.098756	0.041017	0.015328	0.296736
Arthrobacter	0.029091	0.019117	0	0	0	0	0.045984	0.024728
Asanoa	0	0	0	0	0	0	0	0.074184
Azoarcus	0.290909	0.210285	0	0	0	0.102543	0	0

Azospirillum	0	0	0.23488	0.75188	0.375272	0	0.199264	0.148368
Bacillus	0.058182	0.019117	0.322959	0	0	0	0.045984	0
Bacteriovorax	0.101818	0.076467	0	0	0.059253	0.14356	0	0.667656
Bacteroides	0.029091	0.076467	0	0	0	0	0	0
Bauldia	0.058182	0	0	0	0	0	0.030656	0
Beggiatoa	0.596364	0.802906	1.996477	0	0	2.255947	1.578786	3.313551
Bellilinea	0.305455	0.458803	0	0	0	0.266612	0.245248	0.098912
Belnapia	0	0	0	0	0.098756	0	0	0
Blastochloris	0.392727	0.363219	0.411039	0	0.197511	0.14356	0.15328	0.074184
Blastopirellula	0.989091	0.42057	2.613036	0	0	2.420016	3.632741	0.74184
Bradyrhizobium	0.290909	0.133818	0	0	7.64369	0.041017	0.045984	0.173096
Brevundimonas	0	0	0	0.802005	0	0	0.015328	0
Brucella	0.072727	0.019117	0	0	0	0.061526	0.045984	0.024728
Bryocella	0.029091	0.019117	0	0	0	0.041017	0	0
Burkholderia	0.290909	0.344102	0	0.551378	1.027059	0.492207	0.183936	0.840752
Byssovorax	0.087273	0.267635	0	0	0.019751	0.14356	0	0.12364
Caldanaerobacter	0	0	0	0	0.118507	0	0	0
Caldilinea	0	0.076467	0	0	0	0.020509	0.030656	0
Caldithrix	0	0	1.056958	0	0	0	0	0
Caloramator	0.029091	0.038234	0	0	0	0	0	0
Candidatus alysiosphaera	0	0	0	0	0	0	0	0.074184
Candidatus desulforudis	0.189091	0.038234	0.05872	0	0	0.082034	0.107296	0.197824
Candidatus koribacter	0.072727	0.038234	0.02936	0	7.090658	0.348646	0.3832	0.24728
Candidatus magnetobacterium	0.290909	0.114701	0.02936	0	0	0.287121	0.061312	0.197824
Candidatus methylomirabilis	0.029091	0.133818	3.640634	0	0	0.225595	0.321888	0.12364
Candidatus nitrosoarchaeum	0.072727	0.076467	0	0	0.079005	0.061526	0	0
Candidatus odyssella	1.163636	1.873447	1.673517	0	0.059253	0.656276	0.3832	0.667656
Candidatus pelagibacter	0	0.019117	0	0	0	0	0	0.049456
Candidatus saccharimonas	0	0.095584	0	0	0.079005	0.020509	0	0
Candidatus solibacter	0.116364	0.038234	0	0	0.375272	0.082034	0	0.074184
Catelliglobosispora	0	0	0	0.501253	0	0	0	0
Catenuloplanes	0	0	0	0.952381	0	0	0	0
Cellvibrio	0.072727	0.019117	0	0	0	0	0.061312	0.049456

Chelatococcus	0	0	0	0	0.414774	0	0	0
Chitinophaga	0	0	0	0	1.323326	0	0	0
Chloroflexus	1.323636	1.586695	0.763359	0.050125	0	1.086957	0.199264	0.544016
Chondromyces	0.174545	0.267635	0	0	0	0.348646	0.030656	0.296736
Chromatium	0.232727	0.573504	0	0	0.059253	0.184578	0.07664	0.222552
Chryseobacterium	0	0	0	0	0.079005	0	0	0
Chryseolinea	0	0.019117	0	0	0	0	0	0.049456
Chrysiogenes	0.058182	0.038234	0	0.250627	0	0.061526	0.030656	0
Clostridium	0.014545	0.076467	0	0	0.908552	0.041017	0.045984	0.049456
Conexibacter	0.029091	0.019117	0.08808	0	0.079005	0.082034	0.183936	0.12364
Corallococcus	0	0	0.11744	0	0	0	0	0.074184
Cosmarium	0	0	0	0	0	0	0.061312	0.049456
Coxiella	0.130909	0.191168	0.08808	0	0	0.205086	0.275904	0.148368
Cupriavidus	0.494545	0.248518	0.08808	0	0.039502	0.369155	0.15328	0.420376
Curvibacter	0.014545	0.114701	0.17616	0.100251	0	0.184578	0	0.197824
Cyanobacterium	0.058182	0.038234	0.2936	0	0	0	0	0.098912
Cycloclasticus	0	0	0	0	0	0	0	0.098912
Cytophaga	0.014545	0.038234	0	0	0	0.102543	0	0
Dechloromonas	0	0	0	0	0	0.041017	0	0.049456
Deferrisoma	0	0.076467	0	0	0	0.020509	0	0.074184
Defluviicoccus	0.043636	0.114701	0.1468	0	0	0	0.030656	0.12364
Dehalococcoides	0.218182	0.095584	0.20552	0	0.138258	0.246103	0.3832	0.074184
Dehalogenimonas	0	0	0.469759	0	0	0	0	0
Denitratisoma	3.578182	3.364557	3.258955	0.150376	0.138258	5.4758	4.767014	3.165183
Derxia	2.036364	3.65131	0.880799	0	0.217262	5.742412	3.540773	4.500495
Desulfatibacillum	0	0	0	0	0.059253	0	0	0
Desulfobacca	0	0	0	0	0	0.020509	0.030656	0
Desulfobacterium	0	0.191168	0	0	0	0.430681	0	0.049456
Desulfocella	0.130909	0	0.08808	0	0	0.041017	0.291232	0.098912
Desulfococcus	0	0	0.440399	0	0	0	0.030656	0.024728
Desulfocurvus	0.029091	0.019117	0	0	0	0	0	0
Desulfofaba	0	0.05735	0	0	0	0	0	0
Desulfomicrobium	0	0.095584	0.02936	0	0	0	0	0

Desulfomonile	0.058182	0	0	0	0	0.102543	0.030656	0.024728
Desulfonatronospira	0.029091	0	0	0	0	0.020509	0.030656	0
Desulfonauticus	0.029091	0	0	0	0	0.041017	0	0.024728
Desulforegula	1.12	1.070541	0.440399	0	0.059253	0.984413	0.536481	1.2364
Desulforhabdus	0.087273	0.114701	0	0	0	0.082034	0	0
Desulfosarcina	0	0.152934	0	0	0	0.020509	0	0.074184
Desulfovibrio	0.261818	0.114701	0.352319	0	0	0.451189	0.475169	0.395648
Desulfovirga	0.16	0.038234	0.469759	0	0.098756	0.082034	0.015328	0.148368
Desulfuromonas	0.232727	0.095584	0.2936	0.050125	0.039502	0.533224	1.532802	0.272008
Devosia	0.16	0.229402	0	0	0.019751	0.020509	0	0
Dictyoglomus	0.029091	0.152934	0.23488	0	0	0.020509	0.030656	0.12364
Dokdonella	0	0	0.02936	0	0.987557	0.061526	0	0
Dongia	0.043636	0.05735	0	0	0.039502	0.14356	0.413857	0.173096
Duganella	0.029091	0.05735	0	0	0	0.020509	0.858369	0.098912
Dyella	0.014545	0.152934	0.05872	0	0	0.020509	0	0.074184
Edaphobacter	0	0	0	0	4.503259	0	0	0
Elusimicrobium	0.087273	0.152934	0	0	0	0.082034	0	0.049456
Enhydrobacter	0.189091	0	0	0	0	0	0	0
Enhygromyxa	0.058182	0.114701	0	0	0	0.082034	0	0.12364
Eubacterium	0.261818	0.152934	0.17616	0	0	0.205086	0.122624	0.173096
Ferribacterium	0.48	0.172051	0.08808	0	0.019751	0.451189	0.413857	0.420376
Ferrimicrobium	0.014545	0.095584	0	0	0.138258	0	0	0
Ferrovum	0.32	0.688205	0.381679	0	0.118507	0.369155	0.199264	0.593472
Ferruginibacter	0.058182	0.344102	0	0	0.118507	0.123052	0	0.024728
Fibrobacter	0.189091	0.210285	0	0	0	0.164069	0.015328	0.346192
Filomicrobium	0.014545	0	0	0	0	0.061526	0	0.024728
Fimbriimonas	0	0	0	0	0.118507	0	0	0
Flavobacterium	0.887273	1.032307	0.08808	0	0.138258	1.230517	0.659105	2.423343
Flexibacter	0.48	0.324986	0	0	0	0.082034	0.091968	0.148368
Flexithrix	0.16	0.114701	0	0	0	0.061526	0.061312	0.098912
Fluviicola	0	0	0	0	0	0.041017	0	0.074184
Fulvimarina	0.16	0.42057	0.05872	0	0	0.041017	0.137952	0.074184
Gallionella	0	0.649971	0	0	0	0.020509	0	0

Geitlerinema	0	0	0	0	0.158009	0	0	0
Gelria	0.058182	0	0.08808	0	0	0.082034	0.168608	0.148368
Gemmata	0	0	0.1468	0	0.059253	0.061526	0.015328	0
Gemmatimonas	2.705455	1.663162	3.669994	0.050125	0.375272	2.80968	3.602085	1.607319
Geoalkalibacter	0.058182	0	0	0	0	0	0.199264	0
Geobacillus	0.203636	0.191168	0.822079	0	0.019751	0.102543	0.30656	0.148368
Geobacter	3.083636	2.217549	2.025837	0.85213	0.237014	2.317473	3.602085	1.48368
Geopsychrobacter	0.16	0.42057	0.528479	0	0	0.184578	0.15328	0.12364
Georgfuchsia	0.116364	0.05735	0	0	0	0.020509	0	0.049456
Geothermobacter	1.003636	0.363219	1.027598	0	0.019751	1.025431	0.965665	1.038576
Geothrix	0.814545	0.726439	0.05872	0	0.118507	0.164069	0.275904	0.074184
Gloeobacter	0.029091	0.019117	0	0	0.414774	0	0	0
Gluconacetobacter	0	0.324986	0	0	0	0	0	0
Grammonema	0	0	0	0	0	0.061526	0	0.024728
Granulicella	0	0	0	0	1.698598	0.082034	0.015328	0.024728
Haliangium	0.101818	0.05735	0	0	0.059253	0.102543	0.061312	0.049456
Haliea	0	0	0	0	0	0.020509	0	0.148368
Haliscomenobacter	0.436364	0.458803	0	0	0	0.246103	0.015328	0.296736
Halomonas	0.101818	0.649971	0	0	0	0.287121	0.107296	0.24728
Halothermothrix	0.101818	0.305869	0.02936	0	0	0.020509	0	0.024728
Heliobacterium	0	0	0	0	0	0	0	0.074184
Herbaspirillum	0.072727	0	0.02936	0	0.079005	0.020509	0.122624	0.024728
Hippea	0.48	0.324986	0.528479	0	0	0.410172	0.291232	0.148368
Hirschia	0	0.114701	0	0	0	0.020509	0	0
Holophaga	1.978182	0.554387	0.616559	0.200501	4.621766	1.168991	0.873697	1.038576
Hyalangium	0	0	0	0	0	0	0	0.074184
Hydrogenophaga	0.014545	0.191168	0.23488	8.521303	0	0.020509	0.107296	0.074184
Hylemonella	0.101818	0.038234	0	0	0	0.14356	0.07664	0.272008
Hyphomicrobium	0.698182	0.726439	0.17616	0.050125	0.158009	0.492207	0.367872	0.321464
Iamia	0	0	0.08808	0.701754	0	0	0	0
Ideonella	0.087273	0.095584	0	0	0.019751	0.14356	0.015328	0.766568
Ignavibacterium	0.087273	0.114701	0	0	0	0.184578	0	0
Isosphaera	0.087273	0.191168	0.381679	0	0	0.205086	0.107296	0.148368

Janthinobacterium	0.029091	0	1.145038	0	0	0.061526	0.045984	0.024728
Kaistia	0.174545	0.038234	0.1468	0	0	0.102543	0.137952	0.074184
Kofleria	0.276364	0.248518	0.23488	0	0.138258	0.225595	0.107296	0.37092
Kouleothrix	0.043636	0	0	0	0	0	0.061312	0.074184
Kribbella	0	0	0	1.152882	0	0	0	0
Legionella	0	0	0.11744	0	0	0	0	0.074184
Lentisphaera	0.072727	0.038234	0.381679	0	0.059253	0.102543	0.122624	0.074184
Leptothrix	0.130909	0.305869	0	8.370927	0.098756	0.635767	0.030656	1.013848
Leucothrix	0.116364	0.458803	0	0	0	0.758819	0.122624	0.766568
Levilinea	0.101818	0.038234	0	0	0	0.020509	0	0.049456
Longilinea	0.087273	0.573504	0	0.902256	0	0.102543	0	0.098912
Luteolibacter	0	0	0	0	0.059253	0	0	0
Lysinimicrobium	0	0	0	0.401003	0	0	0	0
Lysobacter	0.261818	0.267635	0.1468	0	0.632036	0.184578	1.210914	0.395648
Maricaulis	0	0.019117	0	0	0.039502	0	0	0
Marinilactibacillus	0	0	0	1.102757	0	0	0	0
Marmoricola	0	0.019117	0	0	0.019751	0	0.061312	0.024728
Massilia	0.101818	0.095584	0.11744	0	0.316018	0.184578	4.245861	0.173096
Meganema	0	0	0	0	0.019751	0	0	0.074184
Mesorhizobium	0.014545	0.095584	0.1468	0	0	0.061526	0.045984	0.098912
Methylibium	3.272727	3.249857	3.846154	1.704261	0.059253	6.193601	6.391784	5.341246
Methylobacillus	0	0.611738	0	0	0	0.020509	0	0
Methylobacter	0.232727	0.363219	0	0	0	0	0	0.12364
Methylobacterium	0	0	0	0	0	0.020509	0	0
Methylocapsa	0.014545	0.076467	0	0	0	0.041017	0	0.148368
Methylocella	0.116364	0.076467	0	0	0.35552	0.348646	0.107296	0
Methylococcus	0.901818	0.401453	0.645919	0	0	0.984413	1.410178	0.420376
Methylocystis	0.043636	0.114701	0	0	0	0	0	0
Methylohalobius	0	0.019117	0	0	0	0.246103	0	0.197824
Methylophilus	0	0.038234	0	0	0	0.082034	0.015328	0.074184
Methylopila	0	0	0	0	0.079005	0	0	0
Methylosarcina	0.043636	0.019117	0	0	0	0	0	0
Methylosinus	1.483636	0.841139	0.587199	0	0.454276	1.107465	0.873697	0.593472

Methylothermus	0.058182	0.114701	0	0	0	0.020509	0	0
Methyloversatilis	0	0	0	0	0	0.061526	0	0
Methylovorus	0.014545	0.248518	0	0	0	0.061526	0	0.074184
Microbulbifer	0.247273	0	0	0	0	0	0	0
Microlunatus	0	0.05735	0	0	0	0.020509	0.045984	0.024728
Micromonospora	0	0	0	0.701754	0	0	0	0
Microvirga	0.189091	0.095584	0.411039	0.150376	0.237014	0.369155	0.122624	0.173096
Modestobacter	0	0	0	0	0.17776	0	0	0
Mucilaginibacter	0	0	0	0	0.750543	0	0	0
Mycobacterium	0	0	0.08808	0	1.402331	0	0	0
Natronocella	0	0	0.23488	0	0	0	0	0
Nesterenkonia	0.029091	0.038234	0	0	0	0	0	0.024728
Nevskia	0	0.038234	0.02936	0	0	0.020509	0.015328	0.024728
Niastella	0.261818	0.554387	0	0	0.079005	0.369155	0.030656	0.445104
Nitratireductor	0.029091	0.324986	0	0	0	0	0	0
Nitrincola	0	0.05735	0	0	0	0.061526	0	0.197824
Nitrosospira	0.043636	0.172051	0	0	0	0.102543	0.122624	0.024728
Nitrosovibrio	0.232727	0.076467	1.027598	0	16.23543	0.246103	0.045984	0
Nitrospina	0	0.076467	0	0	0	0.020509	0	0.024728
Nitrospira	6.152727	6.09826	16.99941	1.954887	0.217262	10.35685	12.15512	15.38081
Nitrospirillum	0	0	0	0	0.059253	0	0	0
Nocardioides	0	0	0.02936	5.66416	0	0	0.061312	0
Novosphingobium	0	0	0	1.002506	0	0	0	0
Oceanibaculum	0	0	0	0	0	0	0	0.074184
Ochrobactrum	0	0.05735	0	0	0	0.123052	0	0.074184
Ohtaekwangia	0.989091	0.592621	0.322959	0	0	0.14356	0.199264	0.395648
Oleiphilus	0.014545	0.076467	0	0	0	0	0.015328	0
Oleomonas	0	0	0	0	0.414774	0	0	0
Oligotropha	0.043636	0.038234	0	0	0	0	0	0.024728
Opitutus	1.047273	1.701396	0	0	3.160182	0.225595	0.061312	0.272008
Owenweeksia	0	0.133818	0	0	0	0.020509	0	0.049456
Panacagrimonas	0.043636	0.05735	0.704639	0	0	0.041017	0.260576	0.098912
Parvibaculum	0.029091	0.210285	0	0	0	0	0	0.024728

Pasteuria	0.014545	0	0.02936	0	0	0	0.07664	0
Pedomicrobium	0.290909	0.095584	0.704639	0	0.019751	0.020509	0.214592	0.098912
Pedosphaera	0.654545	0.038234	0.08808	0	0	0.164069	0.07664	0.148368
Pelagibius	0.014545	0.05735	0	0	0	0	0	0
Pelobacter	0.029091	0.076467	0.05872	0	0.059253	0.041017	0.030656	0.222552
Pelomonas	0	0	0	0	0	0	0.275904	0
Pelosinus	0.043636	0	0	0	0	0	0	0
Pelotomaculum	0	0	0.26424	0	0	0	0.045984	0
Petrimonas	0.218182	0.267635	0	0	0	0.205086	0.045984	0.024728
Phaselicystis	0	0	0	0	0.059253	0	0	0
Phenylobacterium	0.276364	0.707322	0.352319	0	0.296267	0.082034	0.3832	0.173096
Phyllobacterium	0	0.05735	0	0	0	0	0	0
Pirellula	1.047273	0.764672	1.526718	0.350877	0.237014	1.148482	1.195586	0.890208
Piscinibacter	0.029091	0	0	0.050125	0	0	0	0.024728
Planctomyces	0.116364	0.133818	0.02936	0	0.138258	0.14356	0.107296	0.395648
Polaromonas	0	0	0.352319	26.16541	0	0	0	0
Polyangium	0.130909	0.191168	0	0	0	0	0.045984	0.049456
Porphyrobacter	0.058182	0.05735	0	0	0	0	0	0.049456
Propionibacterium	0	0	0	1.152882	0	0	0	0
Prosthecobacter	0.64	0.344102	0.05872	0	0.197511	0.246103	0.030656	0.24728
Prosthecomicrobium	0.349091	0.611738	0.17616	0	0.039502	0.102543	0.015328	0.049456
Proteiniphilum	0.014545	0.114701	0.05872	0	0	0.184578	0.045984	0
Pseudanabaena	0.014545	0.05735	0	0	0.059253	0.041017	0.015328	0.049456
Pseudolabrys	0	0.248518	0	0	0.454276	0.020509	0	0
Pseudomonas	1.12	0.994074	3.200235	0.85213	0.276516	0.88187	1.164929	0.914936
Pseudonocardia	0	0	0	3.057644	0	0	0	0
Pseudoxanthomonas	0.014545	0.095584	0	0	0	0.041017	0	0.148368
Ramlibacter	0	0	0	0.250627	0.019751	0	0	0
Reyranella	0.087273	0.133818	0	0.100251	0.237014	0.102543	0	0.24728
Rhizobacter	0.494545	1.204359	0.11744	1.60401	0	1.292043	0.521153	1.533136
Rhizobium	0.378182	0.497037	0.05872	0	8.078214	0.553733	0.045984	0.148368
Rhodanobacter	0	0	0	0	0.197511	0	0	0
Rhodobacter	0	0	0	0	0.079005	0	0	0

Rhodobium	0.014545	0.095584	0.23488	0	0.296267	0.082034	0.015328	0
Rhodocista	0	0	0	0	0.059253	0.061526	0	0
Rhodococcus	0	0	0	0	0	0	0.045984	0
Rhodocyclus	0	0.038234	0.02936	0	0	0.082034	0	0.296736
Rhodoferax	0.261818	0.248518	0.02936	0	0	0.061526	0.137952	0.12364
Rhodomicrobium	0.523636	0.439686	0.08808	0	0.098756	0.020509	0.045984	0.321464
Rhodopila	0	0	0	0	0.197511	0	0	0
Rhodoplanes	1.949091	2.217549	0.08808	0	2.547897	0.410172	0.30656	0.98912
Rhodopseudomonas	0.043636	0.114701	0	0	0.019751	0	0.030656	0
Rhodothermus	0.087273	0.095584	0.02936	0	1.461584	0.041017	0.030656	0
Rhodovastum	0	0	0	0	0.138258	0	0	0
Rickettsia	0	0	0	0	0.079005	0	0	0
Rickettsiella	0	0	0	0	0.079005	0	0	0
Roseomonas	0.101818	0.401453	0.1468	0	0	0.041017	0.030656	0.272008
Rubrivivax	0.029091	0.038234	0	1.102757	0	0.020509	0	0.12364
Rubrobacter	0.247273	0.152934	0.23488	0	0	0.020509	0.091968	0.074184
Runella	1.090909	1.185242	0	0	0	0.697293	0.091968	0.49456
Saccharibacter	0	0.095584	0	0	0.059253	0	0	0
Salicola	0.087273	0.401453	0.02936	0	0	0.082034	0.015328	0.296736
Schlegelella	0.349091	0.535271	0.20552	0	0.098756	0.697293	0.275904	1.038576
Schlesneria	0	0	0	0	0	0.061526	0.015328	0
Shewanella	0	0	0	0	0.17776	0.061526	0	0.074184
Sideroxydans	0	0.152934	0	0	0	0	0	0.024728
Simkania	0.043636	0.114701	0.02936	0	0	0.041017	0	0.074184
Singulisphaera	0	0	0.02936	0	0.197511	0	0	0
Skermanella	0.349091	0.019117	0.1468	0.300752	0.296267	0.020509	0.015328	0.024728
Smithella	0.290909	0.229402	1.233118	0	0.079005	1.066448	1.256898	0.37092
Solimonas	0.029091	0	0.17616	3.408521	0.059253	0	0.061312	0.024728
Solirubrobacter	0	0	0	0	0.316018	0	0	0
Sorangium	0.145455	0.133818	0	0	0	0.164069	0.030656	0.148368
Sphingobacterium	1.469091	1.644045	0.322959	0	1.619593	0.73831	0.597793	1.137488
Sphingomonas	0.130909	0.133818	0	0.100251	0	0.020509	0.15328	0.148368
Sphingopyxis	0	0.019117	0	0.952381	0	0	0	0.049456

Sphingosinicella	0.014545	0	0.05872	0	0.35552	0	0.107296	0.173096
Sphingoterrabacterium	0	0	0	0	0.079005	0	0	0
Spirochaeta	0.014545	0	0	0	0.059253	0.041017	0	0
Spirosoma	0	0.152934	0	0	0	0.041017	0	0
Spirulina	0.101818	0.516154	0.02936	0	0	0.246103	0.061312	0.098912
Spongiibacter	0	0	0	0	0	0.082034	0	0.024728
Sporichthya	0	0	0	0	0	0.102543	0	0
Sporobacterium	0.058182	0.210285	0.11744	0	0.158009	0.041017	0	0
Sporocytophaga	0.276364	0.382336	0	0	0	0.348646	0.030656	0.148368
Stella	0.145455	0.248518	0.352319	0	2.903417	0.041017	0.291232	0.049456
Steroidobacter	0.829091	2.351367	0.587199	0	2.96267	1.086957	0.367872	1.310584
Sterolibacterium	0.32	0.382336	0.499119	0.551378	0.019751	0.369155	0.199264	0.395648
Stigmatella	0.029091	0.89849	0	0	0.118507	0.123052	0.015328	0.074184
Streptococcus	0	0	0	0.150376	0	0	0	0
Streptomyces	0.029091	0.019117	0	0.501253	0	0.205086	0.22992	0
Sulfitobacter	0.145455	0.191168	0	0	0	0.061526	0.015328	0.074184
Sulfurospirillum	0	0.05735	0	0	0	0.020509	0	0
Synechococcus	0	0	0	0	0.059253	0	0	0
Syntrophobacter	0.043636	0.114701	0.08808	0	0	0	0	0
Syntrophorhabdus	0	0.076467	0	0	0	0	0	0
Telmatospirillum	0	0	0	0	0.138258	0	0	0
Terriglobus	0.072727	0.076467	0	0	0.493778	0.123052	0.015328	0.12364
Terrimonas	0.625455	0.191168	0.05872	0	0	0.307629	0.30656	0.346192
Thalassiosira	0	0	0	0	0	0.225595	0	0.049456
Thermaerobacter	0	0	0	0.501253	0	0	0	0
Thermanaeromonas	0.087273	0.152934	0.1468	0	0	0.082034	0.536481	0.272008
Thermanaerothrix	0	0	0	0	0	0.123052	0	0
Thermoanaerobacter	0	0	0.02936	0	0.079005	0.020509	0.091968	0.049456
Thermoanaerobacterium	0	0.038234	0	0	0.059253	0	0	0.074184
Thermobaculum	0.058182	0	0	0	0	0.061526	0.045984	0.098912
Thermodesulfobacterium	0.378182	0.019117	0.02936	0	0	0.102543	0.199264	0.12364
Thermodesulfobium	0.087273	0	0	0	0	0.061526	0.015328	0.074184
Thermodesulforhabdus	0.014545	0	0.11744	0	0	0	0.015328	0.024728

Thermoflavimicrobium	0	0	0	0	0.256765	0	0	0
Thermolithobacter	0.087273	0.05735	0.469759	0	0	0.102543	0.030656	0
Thermomicrobium	0.087273	0	0.17616	0	0	0	0.15328	0
Thermotoga	0.029091	0.038234	0.02936	0	0	0.164069	0.045984	0
Thermus	0.043636	0	0.11744	0	0	0	0.137952	0.024728
Thioalkalivibrio	0	0.019117	0.08808	0	0	0	0	0
Thiobacillus	0	0.267635	0	0	0	0.041017	0.015328	0.173096
Thiobacter	1.541818	1.873447	0.352319	0	0.256765	1.92781	0.812385	2.423343
Thiococcus	0	0.038234	0	0	0	0	0	0.024728
Thiohalomonas	0	0	0	1.553885	0	0	0	0
Thiorhodospira	5.469091	4.033646	2.407516	1.704261	0	5.004102	3.832005	3.264095
Tistrella	0.887273	1.070541	0.528479	0	0	0.492207	0.643777	1.36004
Truepera	0.043636	0.114701	0	0	0	0.020509	0	0.049456
Tumebacillus	0	0	0	0	0.079005	0	0	0
Vampirovibrio	0	0	0	0	0	0.020509	0.07664	0
Variovorax	0.043636	0.038234	0.2936	0.551378	0.138258	0.369155	0.168608	0.296736
Verrucomicrobium	0.058182	0	0.322959	0	0	0	0	0
Vibrio	0.058182	0.152934	0.20552	0	0	0.041017	0.30656	0
Victivallis	0.189091	0.095584	0	0	0	0.061526	0.107296	0.321464
Wolinella	0.276364	0.152934	0.2936	0	0	0.061526	0.321888	0.074184
Xanthomonas	3.229091	1.758746	0.528479	0	0.059253	3.40443	3.494788	1.186944
Zoogloea	0.16	0.095584	0	0	0	0.020509	0	0
