# Supplementary material for: Metabolic traits of sediment bacteria in karst caves in the light of environmental changes
Source: Front Microbiol. 2025 Dec 12;16:1724116. doi: 10.3389/fmicb.2025.1724116 (PMC12742472; doi:10.3389/fmicb.2025.1724116)
Supplement: Supplementary file 4 [file Table_4.PDF]

Supplementary table 4: Utilization of different substrates by microbial communities in sediment samples at 30 °C and under different cultivation conditions (aerobic, anaerobic, anaerobic–aerobic) (Table 1). The threshold for positive readings was set at $OD_{590} \geq 0.400$ and indicated by green shading.

30 °C		Aerobic								Anaerobic								Anaerobic–aerobic							
**Code**	**Substrate**	**S1**	**S2**	**S3**	**S4**	**S5**	**S6**	**S7**	**S8**	**S1**	**S2**	**S3**	**S4**	**S5**	**S6**	**S7**	**S8**	**S1**	**S2**	**S3**	**S4**	**S5**	**S6**	**S7**	**S8**
A1	water	0.00	0.00	0.00	0.00	0.00	0.00	0.00	0.00	0.00	0.00	0.00	0.00	0.00	0.00	0.00	0.00	0.00	0.00	0.00	0.00	0.00	0.00	0.00	0.00
A2	β-methyl-D-glucoside	1.52	0.76	2.52	0.98	2.26	1.45	1.86	2.37	0.18	0.03	0.04	0.05	0.00	0.10	0.08	0.22	1.85	1.11	1.14	0.86	1.19	1.43	2.19	1.97
A3	D-galactonic acid γ-lactone	1.15	1.26	2.16	1.00	1.77	2.21	1.64	1.93	0.00	0.12	0.16	0.06	0.00	0.01	0.00	0.00	0.67	1.26	1.75	0.00	1.67	2.20	1.90	2.04
A4	L-arginine	1.15	0.74	1.15	0.67	0.88	1.85	1.15	1.33	0.01	0.00	0.02	0.00	0.00	0.08	0.00	0.03	0.89	0.94	2.12	1.00	1.38	1.75	1.05	1.94
B1	pyruvic acid methyl ester	0.84	0.36	1.62	0.07	1.49	1.13	1.33	0.65	0.01	0.04	0.03	0.14	0.00	0.00	0.00	0.01	0.29	0.95	0.10	0.78	0.00	0.27	1.08	0.81
B2	D-xylose	1.16	1.04	1.16	0.40	1.05	1.92	0.73	1.62	0.00	0.01	0.06	0.05	0.14	0.00	0.43	0.09	0.05	0.35	0.66	0.73	0.87	0.36	0.91	1.05
B3	D-galacturonic Acid	1.79	0.86	1.98	0.18	1.60	0.51	2.34	1.60	0.00	0.01	0.02	0.04	0.19	0.03	0.00	0.04	0.27	0.17	0.20	0.28	0.99	0.77	1.18	1.00
B4	L-asparagine	1.83	0.59	2.01	0.23	1.40	1.40	2.25	1.51	0.00	0.04	0.04	0.09	0.03	0.13	0.00	0.11	0.41	0.32	0.38	0.32	0.81	0.73	1.36	0.99
C1	Tween 40	1.32	0.62	2.81	0.18	2.29	1.46	1.56	2.25	0.00	0.02	0.03	0.05	0.39	0.23	0.00	0.14	0.18	0.08	0.27	0.00	0.51	0.35	0.76	1.03
C2	i-erythritol	0.62	0.01	1.01	0.02	2.24	1.01	0.46	0.30	1.58	1.10	1.23	0.35	0.94	1.21	1.07	0.80	1.40	1.28	1.01	0.27	1.53	1.21	1.68	1.58
C3	2-hydroxy benzoic acid	1.19	0.70	1.32	0.18	1.76	2.21	1.43	0.83	0.03	0.14	0.06	0.02	0.60	0.00	0.00	0.08	0.65	0.19	0.35	0.28	1.81	0.92	1.11	1.68
C4	L-phenylalanine	2.94	1.73	2.88	1.01	2.42	2.67	2.38	2.09	0.00	0.06	0.06	0.09	1.02	0.21	0.00	0.06	1.01	0.33	2.51	1.33	2.01	1.66	2.60	1.88
D1	Tween 80	2.33	0.95	2.61	0.42	2.12	2.30	2.78	2.29	0.03	0.03	0.04	0.06	0.00	0.18	0.02	0.25	1.26	0.08	1.68	0.66	1.45	1.13	2.15	1.62
D2	D-mannitol	2.70	0.16	2.75	0.55	1.87	2.36	1.46	2.12	0.01	0.05	0.04	0.01	0.11	0.00	0.03	0.03	0.85	2.32	1.88	0.67	1.66	0.98	0.42	1.91
D3	4-hydroxy benzoic acid	1.17	0.17	1.58	0.38	1.97	0.88	0.86	1.33	0.02	0.02	0.05	0.09	0.26	0.19	0.03	0.02	0.45	1.07	0.22	1.08	0.92	1.13	1.08	0.67
D4	L-serine	0.83	0.14	1.87	0.38	1.17	1.67	1.70	1.61	0.14	0.10	0.03	0.04	0.01	0.03	0.02	0.06	0.59	0.77	0.43	0.98	0.82	0.28	1.02	0.60
E1	α-cyclodextrin	1.75	0.58	2.39	0.35	2.39	1.03	1.33	1.92	0.00	0.02	0.16	0.04	0.08	0.01	0.00	0.01	0.33	0.02	1.15	0.10	0.96	1.32	1.05	1.87
E2	N-acetyl-D-glucosamine	1.02	0.89	2.54	0.08	2.17	2.92	2.25	2.48	0.03	0.07	0.48	0.00	0.09	0.02	0.05	0.07	0.91	1.64	1.64	1.21	1.90	0.88	1.91	1.11
E3	γ-hydroxybutyric acid	0.11	0.02	0.53	0.01	0.20	0.41	0.03	0.01	0.02	0.04	0.03	0.00	0.06	0.02	0.05	0.02	0.00	0.04	0.04	0.00	0.05	1.49	0.03	0.24
E4	L-threonine	0.90	0.46	2.60	0.07	1.91	2.45	2.00	2.40	0.00	0.04	0.04	0.07	0.16	0.00	0.02	0.02	0.72	0.73	1.28	0.48	0.93	1.34	2.00	1.85
F1	glycogen	1.18	0.66	1.82	0.22	0.91	1.27	1.26	0.74	0.00	0.02	0.13	0.07	0.00	0.00	0.03	0.01	0.07	0.21	0.57	0.19	0.21	0.32	1.01	0.96
F2	D-glucosaminic acid	0.67	0.08	2.61	0.33	1.07	0.87	1.30	0.51	0.00	0.01	0.01	0.05	0.08	0.00	0.01	0.01	0.00	0.76	0.49	0.24	0.38	1.14	1.19	1.05
F3	itaconic acid	0.18	0.01	0.04	0.01	0.26	0.11	0.04	0.96	0.02	0.18	0.03	0.02	0.00	0.00	0.00	0.02	0.14	0.02	0.03	0.00	0.16	0.01	0.00	0.07
F4	glycyl-L-glutamic acid	2.04	1.04	2.34	0.04	1.46	1.54	1.99	2.29	0.00	0.01	0.15	0.01	0.00	0.02	0.05	0.02	0.96	0.37	1.21	0.56	0.38	0.59	1.02	1.59
G1	D-cellobiose	2.84	1.95	2.81	0.76	2.63	2.68	2.04	2.87	0.00	0.02	0.22	0.04	0.06	0.00	0.00	0.01	0.92	0.44	2.12	1.02	1.59	1.56	2.05	2.89
G2	glucose-1-phosphate	3.13	0.88	2.93	0.52	2.39	1.81	2.95	2.78	0.01	0.02	0.00	0.04	0.01	0.00	0.01	0.03	1.92	0.99	2.40	1.31	2.41	2.61	2.72	2.63
G3	α-ketobutyric acid	1.73	0.45	2.47	0.31	1.81	2.21	1.03	2.20	0.01	0.02	0.04	0.06	0.04	0.00	0.04	0.06	0.61	0.33	0.45	0.30	0.86	1.19	0.85	1.65
G4	phenylethyl-amine	2.79	0.02	2.77	0.61	2.27	2.65	2.83	2.50	0.00	0.03	0.06	0.05	0.16	0.03	0.13	0.02	1.21	0.86	2.32	0.62	1.92	1.87	2.29	2.12
H1	α-D-lactose	1.06	0.69	2.34	0.24	1.02	1.70	1.38	1.10	0.00	0.03	0.15	0.10	0.09	0.07	0.02	0.04	0.40	0.13	1.94	0.46	0.71	1.36	1.38	0.25
H2	D, L-α-glycerol phosphate	1.59	0.11	2.66	0.22	1.36	2.45	1.66	1.85	0.00	0.01	0.06	0.07	0.02	0.00	0.01	0.09	0.78	0.51	0.90	0.37	1.08	0.89	1.26	1.13
H3	D-malic acid	0.65	0.23	1.58	0.06	1.16	1.18	0.96	1.01	0.02	0.03	0.12	0.08	0.02	0.03	0.03	0.10	0.96	0.03	1.18	0.64	0.45	0.57	1.28	0.95
H4	putrescine	1.56	0.71	2.43	0.00	1.24	1.79	2.45	2.20	0.00	0.00	0.04	0.08	0.00	0.05	0.03	0.13	0.38	0.36	1.36	0.68	1.20	0.92	1.89	1.69
**AMR**		**1.48**	**0.61**	**2.07**	**0.34**	**1.63**	**1.68**	**1.59**	**1.67**	**0.07**	**0.07**	**0.12**	**0.06**	**0.15**	**0.09**	**0.07**	**0.08**	**0.68**	**0.60**	**1.09**	**0.56**	**1.06**	**1.07**	**1.37**	**1.38**
**CMD**		**93.5**	**64.5**	**96.8**	**29.0**	**93.5**	**96.8**	**93.5**	**93.5**	**3.2**	**3.2**	**6.5**	**0.0**	**9.7**	**3.2**	**6.5**	**3.2**	**67.7**	**48.4**	**74.2**	**58.1**	**80.6**	**80.6**	**93.5**	**90.3**
