# Supplementary material for: Metabolic traits of sediment bacteria in karst caves in the light of environmental changes
Source: Front Microbiol. 2025 Dec 12;16:1724116. doi: 10.3389/fmicb.2025.1724116 (PMC12742472; doi:10.3389/fmicb.2025.1724116)
Supplement: Supplementary file 5 [file Table_5.PDF]

Supplementary table 5: Percentage of utilization of different substrates in seven cave sediments (samples excluding S5 – karst soil) by microbial communities at different temperature (10 °C, 20 °C, 30 °C) and oxygen cultivation conditions (aerobic, anaerobic–aerobic). Green shading indicates < 50% metabolized substrates in the cave samples.

| | | 10 °C | | 20 °C | | 30 °C | |
|---|---|---|---|---|---|---|---|
| | | Aerobic (%) | Anaerobic–aerobic (%) | Aerobic (%) | Anaerobic–aerobic (%) | Aerobic (%) | Anaerobic–aerobic (%) |
| A1 | water | 0.0 | 0.0 | 0.0 | 0.0 | 0.0 | 0.0 |
| A2 | β-methyl-D-glucoside | 71.4 | 85.7 | 100.0 | 100.0 | 100.0 | 100.0 |
| A3 | D-galactonic acid γ-lactone | 100.0 | 100.0 | 100.0 | 100.0 | 100.0 | 85.7 |
| A4 | L-arginine | 100.0 | 100.0 | 100.0 | 100.0 | 100.0 | 100.0 |
| B1 | pyruvic acid methyl ester | 71.4 | 0.0 | 85.7 | 14.3 | 71.4 | 57.1 |
| B2 | D-xylose | 71.4 | 28.6 | 100.0 | 28.6 | 85.7 | 57.1 |
| B3 | D-galacturonic Acid | 42.9 | 42.9 | 85.7 | 57.1 | 85.7 | 42.9 |
| B4 | L-asparagine | 71.4 | 0.0 | 85.7 | 57.1 | 85.7 | 57.1 |
| C1 | Tween 40 | 85.7 | 28.6 | 100.0 | 42.9 | 85.7 | 28.6 |
| C2 | i-erythritol | 14.3 | 28.6 | 71.4 | 57.1 | 57.1 | 85.7 |
| C3 | 2-hydroxy benzoic acid | 57.1 | 28.6 | 100.0 | 14.3 | 85.7 | 57.1 |
| C4 | L-phenylalanine | 85.7 | 100.0 | 100.0 | 100.0 | 100.0 | 85.7 |
| D1 | Tween 80 | 85.7 | 85.7 | 100.0 | 100.0 | 100.0 | 85.7 |
| D2 | D-mannitol | 85.7 | 85.7 | 85.7 | 85.7 | 85.7 | 100.0 |
| D3 | 4-hydroxy benzoic acid | 57.1 | 28.6 | 85.7 | 14.3 | 71.4 | 85.7 |
| D4 | L-serine | 71.4 | 0.0 | 100.0 | 28.6 | 71.4 | 85.7 |
| E1 | α-cyclodextrin | 85.7 | 100.0 | 100.0 | 85.7 | 85.7 | 57.1 |
| E2 | N-acetyl-D-glucosamine | 85.7 | 100.0 | 85.7 | 85.7 | 85.7 | 100.0 |
| E3 | γ-hydroxybutyric acid | 0.0 | 14.3 | 42.9 | 28.6 | 28.6 | 14.3 |
| E4 | L-threonine | 100.0 | 100.0 | 100.0 | 100.0 | 85.7 | 100.0 |
| F1 | glycogen | 57.1 | 0.0 | 85.7 | 0.0 | 85.7 | 42.9 |
| F2 | D-glucosaminic acid | 57.1 | 57.1 | 85.7 | 57.1 | 71.4 | 71.4 |
| F3 | itaconic acid | 14.3 | 0.0 | 28.6 | 14.3 | 14.3 | 0.0 |
| F4 | glycyl-L-glutamic acid | 57.1 | 100.0 | 100.0 | 100.0 | 85.7 | 85.7 |
| G1 | D-cellobiose | 100.0 | 71.4 | 85.7 | 71.4 | 100.0 | 100.0 |
| G2 | glucose-1-phosphate | 100.0 | 85.7 | 100.0 | 100.0 | 100.0 | 100.0 |
| G3 | α-ketobutyric acid | 71.4 | 14.3 | 100.0 | 14.3 | 85.7 | 71.4 |
| G4 | phenylethyl-amine | 71.4 | 100.0 | 100.0 | 71.4 | 85.7 | 100.0 |
| H1 | α-D-lactose | 42.9 | 85.7 | 100.0 | 100.0 | 85.7 | 71.4 |
| H2 | D, L-α-glycerol phosphate | 71.4 | 85.7 | 100.0 | 85.7 | 71.4 | 85.7 |
| H3 | D-malic acid | 71.4 | 100.0 | 85.7 | 85.7 | 71.4 | 85.7 |
| H4 | putrescine | 85.7 | 100.0 | 100.0 | 100.0 | 85.7 | 71.4 |
