# Supplementary material for: Metabolic traits of sediment bacteria in karst caves in the light of environmental changes
Source: Front Microbiol. 2025 Dec 12;16:1724116. doi: 10.3389/fmicb.2025.1724116 (PMC12742472; doi:10.3389/fmicb.2025.1724116)
Supplement: Supplementary file 6 [file Image_1.PDF]

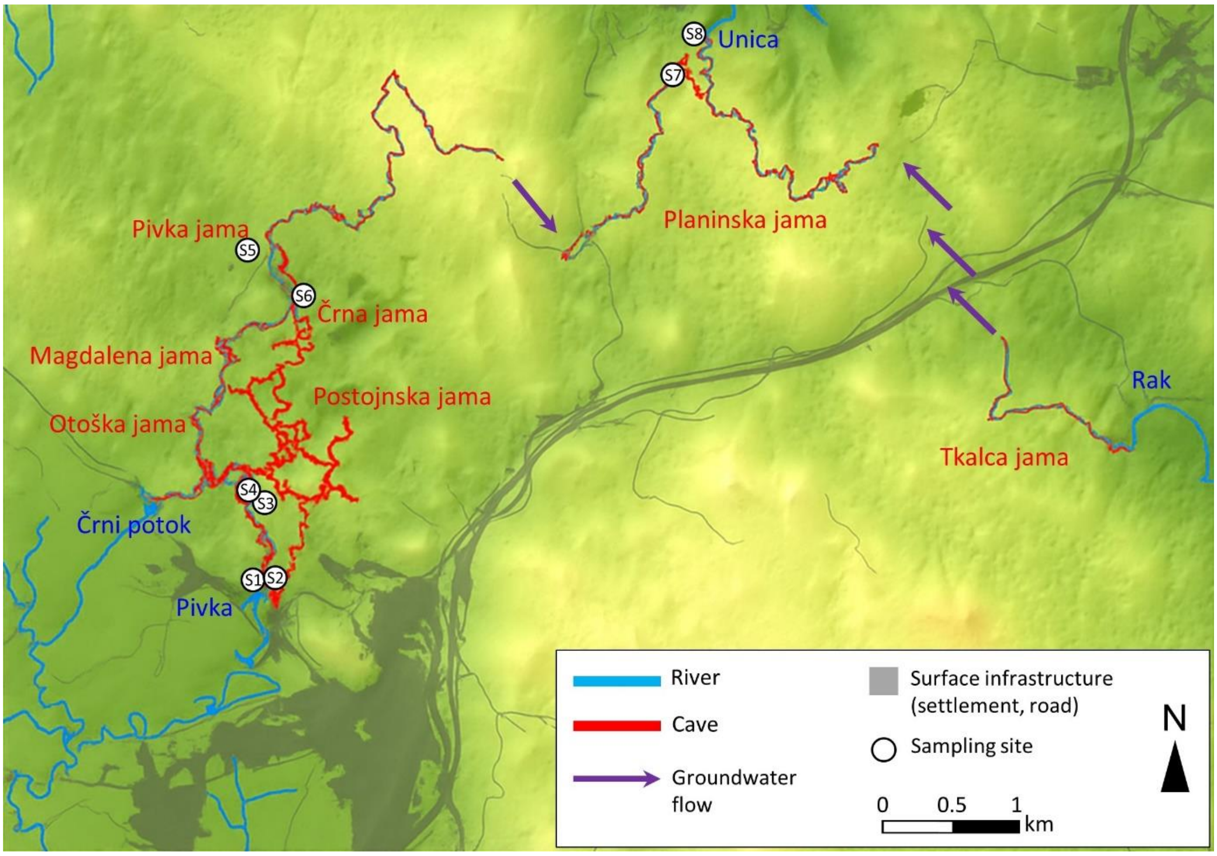

Supplementary figure 1: Map of the area with sampling sites (based on Slovenian Environment Agency, Lidar data fishnet, available from: http://gis.arso.gov.si/, and Cave Registry of the Karst Research Institute ZRC SAZU and Speleological Association of Slovenia)
