# Supplementary figures and images for: Metabolic traits of sediment bacteria in karst caves in the light of environmental changes
Source: Front Microbiol. 2025 Dec 12;16:1724116. doi: 10.3389/fmicb.2025.1724116 (PMC12742472; doi:10.3389/fmicb.2025.1724116)

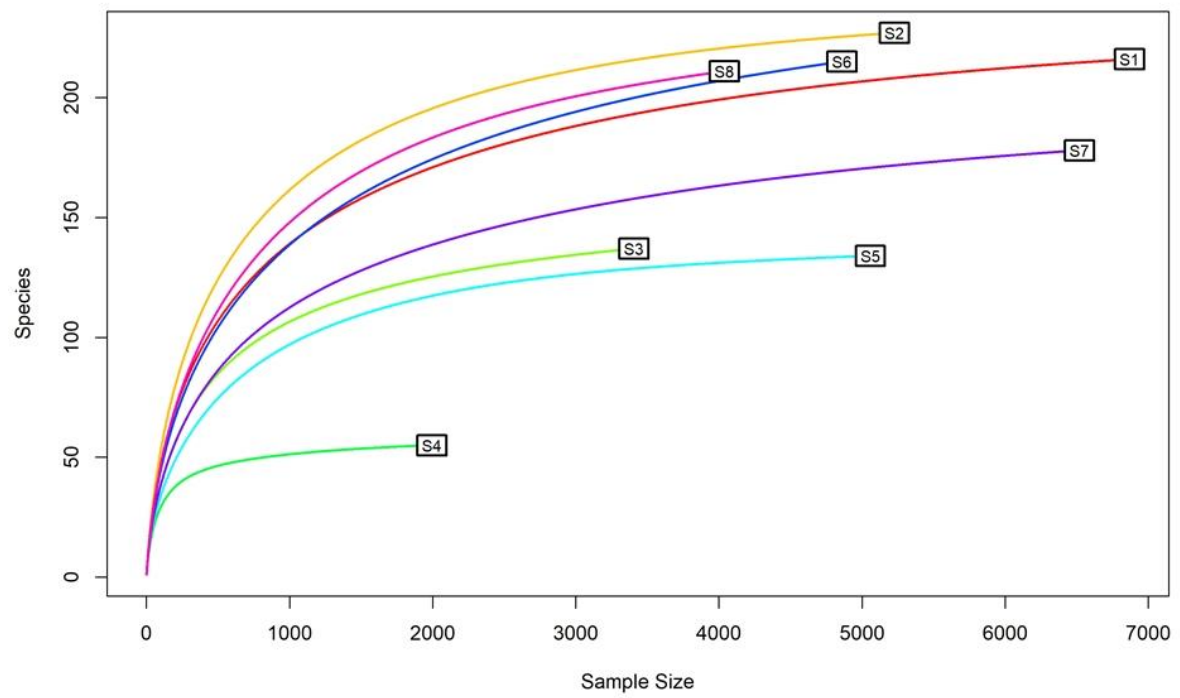

Supplementary figure 2: Rarefaction curves for Bacteria.

Supplement: Supplementary file 7 [file Image_2.PDF]
