# Supplementary material for: Metabolic traits of sediment bacteria in karst caves in the light of environmental changes
Source: Front Microbiol. 2025 Dec 12;16:1724116. doi: 10.3389/fmicb.2025.1724116 (PMC12742472; doi:10.3389/fmicb.2025.1724116)
Supplement: Supplementary file 8 [file Image_3.PDF]

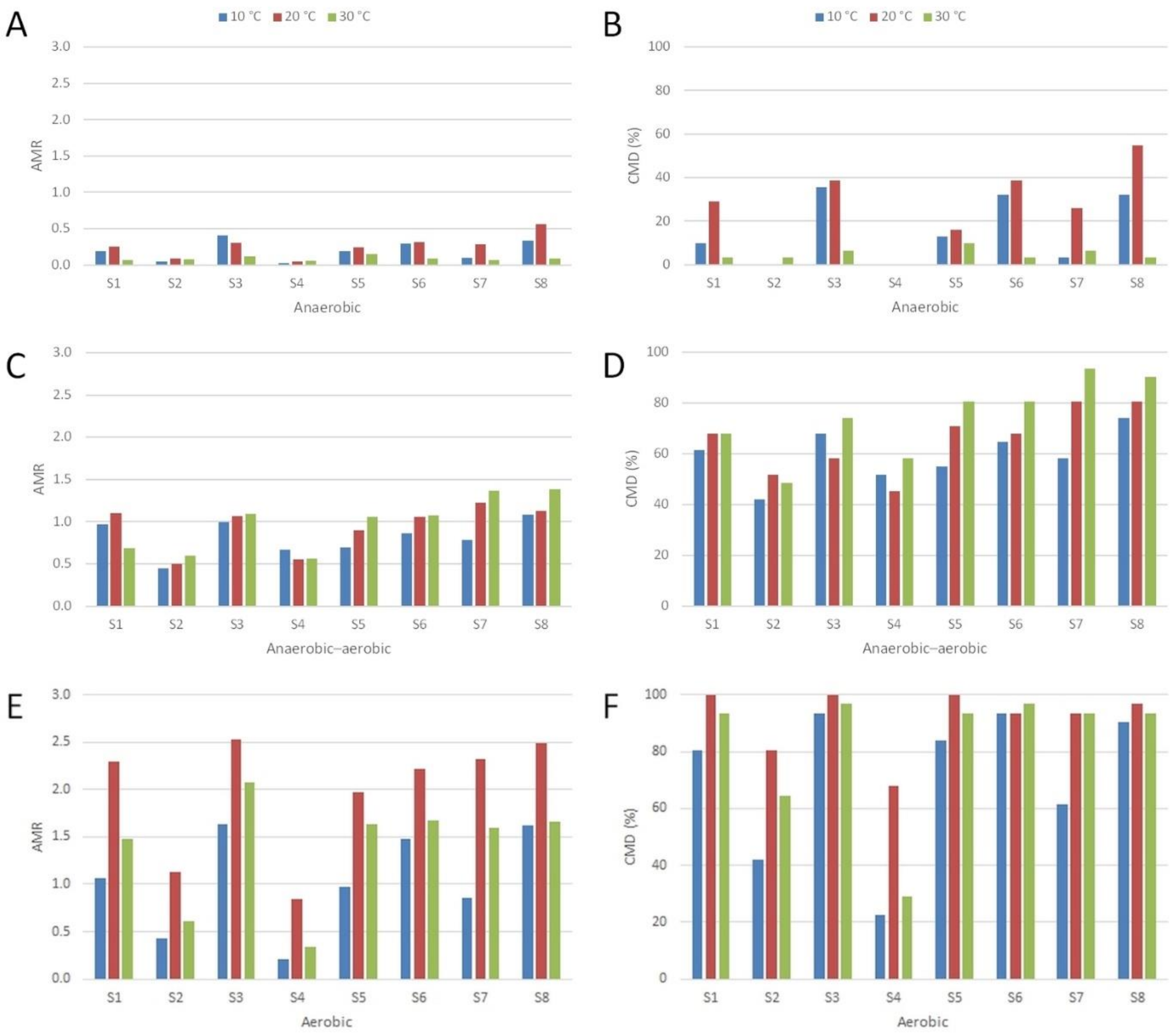

Supplementary figure 3: CLPP profiles for samples (S1 – S8). A, C, E: average metabolic response (AMR) at different temperatures (10 °C, 20 °C, 30 °C) and oxygen availability (anaerobic, anaerobic–aerobic, aerobic). B, D, F: community metabolic diversity (CMD) at different temperatures (10 °C, 20 °C, 30 °C) and oxygen availability (anaerobic, anaerobic–aerobic, aerobic).
